# Integrative study of two invasive North American pine aphids (*Cinara atlantica* and *C. watsoni*) in South Korea

**DOI:** 10.1038/s41598-026-46921-z

**Published:** 2026-05-17

**Authors:** Minho Lee, Mariusz Kanturski, Colin Favret, Seunghwan Lee

**Affiliations:** 1 Department of Agricultural Biotechnology Insect Biosystematics Laboratory , Seoul National University , 08826 Seoul, Korea; 2 Research Institute of Agricultural and Life Sciences , Seoul National University , Seoul, 08826 Korea; 3 Institute of Biology, Biotechnology and Environmental Protection Faculty of Natural Sciences , University of Silesia , Bankowa 9, Katowice, 40-007 Poland; 4 Department of Biological Sciences , Université de Montréal , 4101 East Sherbrooke Street, Montréal, QC H1X 2B2 Canada

**Keywords:** Adventive aphid, Morph, Life cycle, Pine forest pest, DNA barcoding, Ecology, Ecology, Evolution, Plant sciences

## Abstract

This study investigates the seasonal ecology, morphology, and invasion history of the aphids *Cinara atlantica* and *C. watsoni* (Hemiptera: Aphididae: Lachninae) in South Korea and assesses their potential ecological impacts. Historically, *C. atlantica* was recorded only on non-native pines, but we provide the first evidence of host expansion to the native pine *Pinus densiflora*. *C. watsoni* is recorded for the first time in South Korea on imported pines, indicating a potential risk of spread to native hosts. Field surveys conducted between 2020 and 2025, including monthly monitoring, showed that both species reproduce anholocyclically, with colony densities peaking in June and October–November. Honeydew accumulation promoted sooty mold development, and heavy infestations caused needle yellowing. Morphological redescriptions, DNA barcoding, and haplotype analyses confirmed species identities and revealed minimal genetic divergence between South Korean and Nearctic populations. Shared haplotypes with North American material suggest a recent introduction possibly mediated by international plant trade, although the precise source remains uncertain. These findings highlight the high invasion potential of *Cinara* species and emphasize the need for strengthened phytosanitary measures and continuous monitoring of conifer-associated aphids in East Asia.

**Supplementary Information:**

The online version contains supplementary material available at 10.1038/s41598-026-46921-z.

## Introduction

Biological invasions pose significant global economic and ecological threats, as invasive species disrupt native communities through predation, competition, and other biotic interactions^1–4^. The rapid increase in international trade and the movement of ornamental plants has accelerated the introduction of non-native organisms across continents^5,6^. Pine forests constitute the largest forest ecosystems in the Northern Hemisphere, extending from subtropical to subarctic regions and covering diverse landscapes including coastal plains, plateaus, and mountains^7^. Pines are widely used for afforestation, accounting for approximately 20% of global plantation forests, due to their commercial value and ecological functions such as soil stabilization and forest restoration^8,9^. In South Korea, native pine species such as *Pinus densiflora*, *P*. *thunbergii*, *P*. *koraiensis*, *P*. *pumila*, and *P*. *parviflora* are ecologically and culturally significant, with *P*. *densiflora* functioning as a keystone species that shapes forest structure and underpins cultural heritage^10–12^. Additionally, several exotic pine species, including *Pinus banksiana*, *P*. *rigida*, *P*. *strobus*, *P*. *sylvestris*, and *P*. *taeda* have been introduced from North America and Europe for afforestation and landscaping^11^.

Aphids (Hemiptera: Aphididae) rank among the most successful invasive insect taxa worldwide, largely due to their parthenogenetic reproduction, broad host range, and phenotypic plasticity, which facilitate rapid population growth and ecological adaptability^13–16^. They damage host plants through direct phloem feeding, transmit phytoplasmas and viruses, and promote sooty mold growth via honeydew secretion, thereby reducing plant vigor and photosynthetic capacity^17–19^. The genus *Cinara* Curtis, 1835, comprises more than 200 described species worldwide^16^, most of which exhibit strong host specificity to coniferous trees, particularly members of the families Cupressaceae and Pinaceae^20–23^. These aphids typically form dense colonies on the stems, branches, and roots of their hosts^24^ and in some cases, act as vectors of plant pathogens such as phytoplasmas, posing additional risks to forest health^18^. The international trade of nursery stock and ornamental conifers has greatly increased the likelihood of intercontinental introductions of *Cinara* species^25^. Among them, *Cinara cupressi* (Buckton, 1881) is listed among the world’s 100 worst invasive alien species and has caused severe damage to Cupressaceae across Africa, Europe, and South America, resulting in economic losses^22,26–30^. These examples highlight the high invasion potential of *Cinara* species and emphasize the importance of monitoring their occurrence. In South Korea, *Cinara* species have been increasingly detected on imported coniferous plants, particularly non-native *Pinus* species, with *C*. *atlantica* (Wilson, 1919), native to North America and the Caribbean, first reported in 1994 on *P*. *rigida* and *P*. *banksiana*^31^. This species is known for its host specificity to multiple *Pinus* species and has been reported from Argentina, Brazil, Cuba, Costa Rica, and Jamaica^16,32–36^. Despite these reports, little is known about the seasonal ecology, host associations, and overwintering strategies of these introduced *Cinara* species in South Korea. Furthermore, their invasion pathways and genetic relationships with populations in their native range remain poorly resolved.

In this study, we report the first record of *Cinara watsoni* Tissot, 1939 from South Korea. This species, native to North America^37–43^, has previously been reported from Central America (Costa Rica) and China^16,35,36,44,45^. Specifically, the objectives of this study are to: (1) present updated taxonomic diagnoses of *C*. *atlantica* and *C*. *watsoni* through detailed morphological redescriptions and ecological illustrations of both apterous and alate viviparous females, (2) perform molecular identification of *C*. *atlantica* and *C*. *watsoni* using mitochondrial COI barcoding and infer their invasion pathways through TCS haplotype network analysis, and (3) investigate the seasonal ecology, host range, and life cycle of both species in South Korea, thereby establishing a foundation for future pest management strategies.

## Results

### *Cinara atlantica *(Wilson, 1919)

#### Apterous viviparous female – re-description

**Fig. 1:**
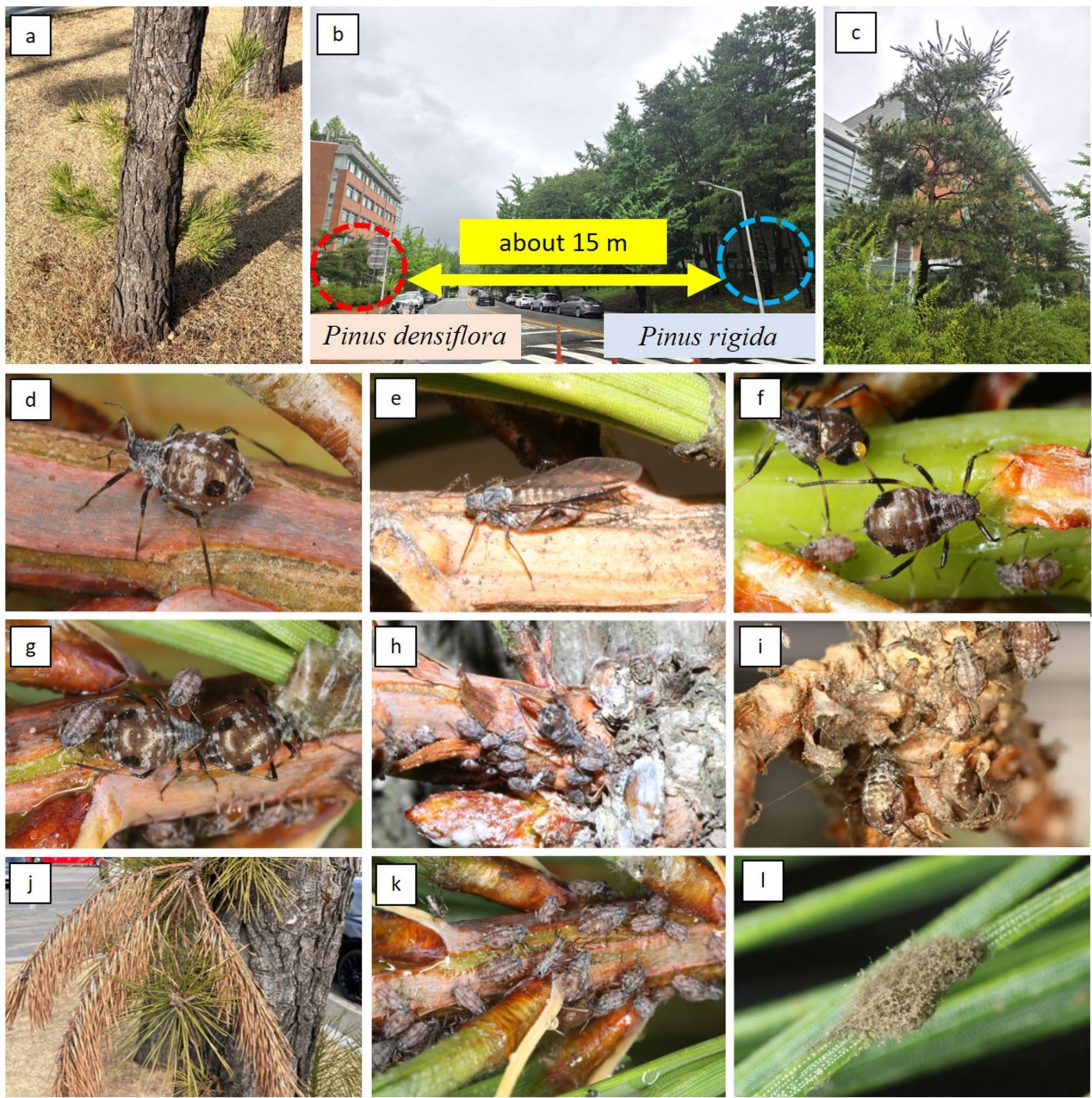
*Cinara atlantica*. In life: (**a**) *Pinus rigida*, (**b**) first record of a colony on the native pine (*P. densiflora*) in South Korea, (**c**) *P. densiflora*, (**d**) apterous viviparous female, (**e**) alate viviparous female, (**f**) colony on a shoot of *P. rigida* (in spring), (g) colony on a young branch of *P. rigida* (in fall), (**h**) colony at the base of branch of *P. rigida* (in winter), (**i**) colony on a young branch of *P. densiflora* (in winter), (**j**) yellowing of the needles of *P. rigida*, (**k**) honeydew produced by aphids, (**l**) aphids causing black sooty mold on needles.

**Fig. 2:**
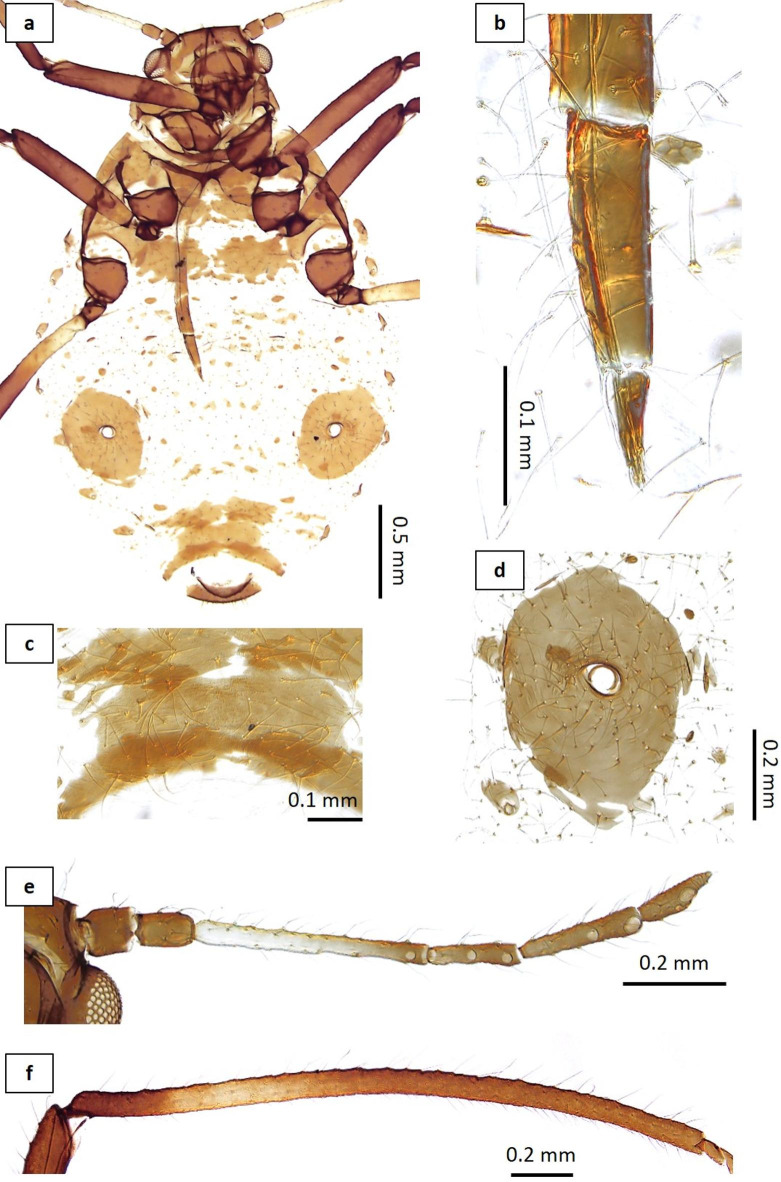
Morphological characters of *Cinara atlantica*, apterous viviparous female (LMHCAAP-2): (**a**) body, (**b**) URS, (**c**) GP, (**d**) SIPH, (**e**) ANT, (**f**) hind tibia

Figures 1d and 2; Table 1.

**Table 1:** Biometric measurement of *Cinara atlantica* (min.–max.) mean length (mm). Measurements from the original description are shown for comparison with newly examined non-type specimens.

Species	*Cinara atlantica* (orig. descr. by Wilson, 1919)		*Cinara* *atlantica*	
Character	apterous viviparous female	alate viviparous female	apterous viviparous female (*n* = 27)	alate viviparous female (*n* = 5)
BL	2.37	2.39	(2.54–3.38) 2.95	(2.69–3.80) 3.18
MaxW	-	-	(1.43–2.22) 1.77	(1.03–1.59) 1.32
HW	-	-	(0.62–0.71) 0.67	(0.62–0.73) 0.66
ANT I	-	-	(0.08–0.11) 0.09	(0.08–0.11) 0.09
ANT II	-	-	(0.09–0.11) 0.10	(0.10–0.12) 0.11
ANT III	0.44	0.46	(0.35–0.48) 0.43	(0.44–0.54) 0.48
ANT IV	0.21	0.21	(0.16–0.23) 0.19	(0.16–0.24) 0.20
ANT V	0.23	0.23	(0.20–0.26) 0.22	(0.21–0.29) 0.23
BASE	-	-	(0.09–0.14) 0.12	(0.11–0.14) 0.12
PT	-	-	(0.04–0.05) 0.04	(0.04–0.05) 0.04
URS	0.187 + 0.063 = 0.25	0.187 + 0.063 = 0.25	(0.25–0.29) 0.26	(0.24–0.27) 0.25
FEMORA III	-	-	(0.96–1.37) 1.18	(1.10–1.48) 1.25
TIBIAE III	2.20	2.08	(1.74–2.37) 2.04	(2.03–2.63) 2.20
HT Ib	-	-	(0.04–0.05) 0.04	(0.04–0.05) 0.04
HT Id	-	-	(0.05–0.06) 0.05	(0.05–0.06) 0.05
HT Iv	-	-	(0.10–0.13) 0.11	(0.11–0.12) 0.11
HT II	-	-	(0.23–0.30) 0.27	(0.26–0.31) 0.27
SIPH cone	-	-	(0.46–0.69) 0.59	(0.46–0.60) 0.52
GP L	-	-	(0.14–0.25) 0.20	(0.18–0.25) 0.21
GP W	-	-	(0.33–0.54) 0.44	(0.38–0.51) 0.43
FWL	-	-	-	(3.55–4.37) 3.89

**Fig. 3:**
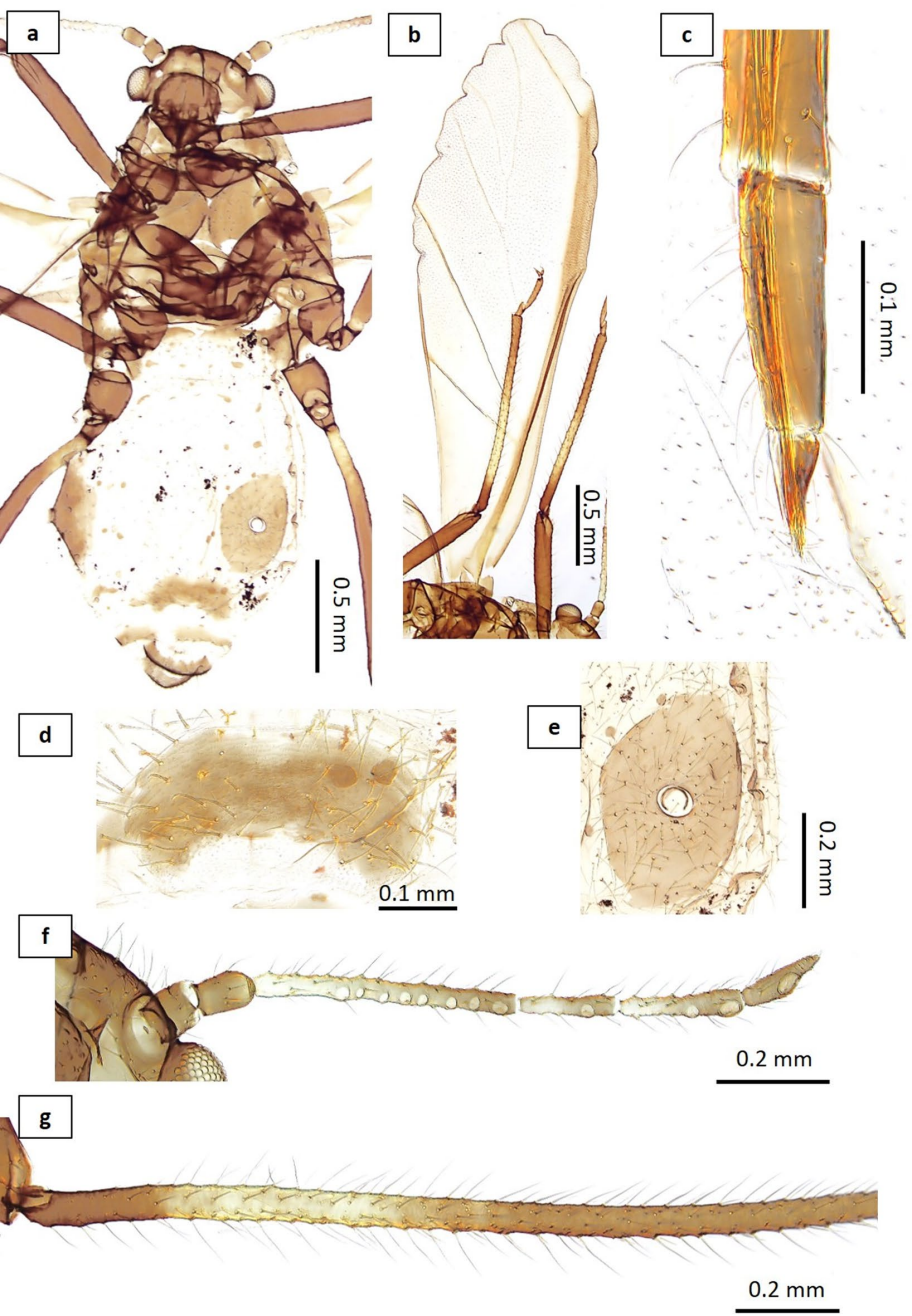
Morphological charactersof*Cinara atlantica*, alate viviparous female (LMHCAAL-1): (**a**) body, (**b**) forewing, (**c**) URS, (**d**) GP, (**e**) SIPH, (**f**) ANT, (**g**) hind tibia

Color. In life: head, prothorax, and mesothorax dark brown. Metathorax and ABD I brown with paired dark brown irregular dorsal patches. ABD II–VII brown. Whole body with slightly greyish-white wax (Fig. 1d). ANT I–II brown, ANT III light brown with 1/3 distal end brown, ANT IV–VI brown. Fore and middle uniformly dark, hind femora dusky brown with light brown basal parts. Fore tibiae with slightly light brown basal parts. Middle and hind tibiae with light brown basal parts. Tarsi brown. SIPH dark brown. Pigmentation in mounted specimens: head, prothorax, and mesothorax brown. Metathorax and ABD I light brown with paired dark brown irregular dorsal patches. ABD II–VII light brown (Fig. 2a). ANT I–II brown, ANT III light brown with 1/3 distal end brown, ANT IV–VI brown (Fig. 2e). Fore and middle uniformly dark, hind femora dusky brown with light brown basal parts. Fore tibiae uniformly dark with slightly light brown basal parts. Middle and hind tibiae uniformly dark with light brown basal parts (Fig. 2f). Tarsi, SIPH, Cauda, and GP brown. Morphometric characters: body elongated oval (Fig. 2a). HW 0.51–0.59 × ANT. ANT 0.37–0.46 × BL. ANT III with 0–2 secondary rhinaria, shorter than ANT IV+V+VI. ANT IV shorter than ANT V with 1–2 secondary rhinaria. ANT V longer than ANT VI with one rounded primary rhinarium with 1–2 small rounded secondary rhinaria. ANT VI with PT 0.29–0.45 × BASE, with one rounded primary rhinarium and 4–5 accessory rhinaria. Other antennal ratios: VI/III 0.30–0.44, V/III 0.44–0.59, IV/III 0.38–0.51. LS ANT III 1.58–2.79 × BD III. Rostrum reaches ABD IV–V. URS 0.54–0.69 × ANT III, 1.44–1.99 × ANT VI and 0.92–1.07 × HT II with 5–8 fine accessory setae (Fig. 2b). HT I basal length 0.62–0.88 × dorsal, 0.33–0.45 × ventral. HT II 0.55–0.71 × ANT III and 1.45–1.88 × ANT VI. ABD VIII in form of broken band and few small scleroites with 11–18 setae. GP U-shaped with 36–51 fine setae (Fig. 2c). SIPH cone 9.22–13.85 × SIPH pore (Fig. 2d). TIBIAE III covered with long fine setae on dorsal side longer (0.08–0.12 mm) than the width of TIBIAE III at midpoint (Fig. 2f). Cauda semi-circular with many long fine, pointed setae.

#### Alate viviparous female – re-description

Figures 1e and 3; Table 1.

**Fig. 4:**
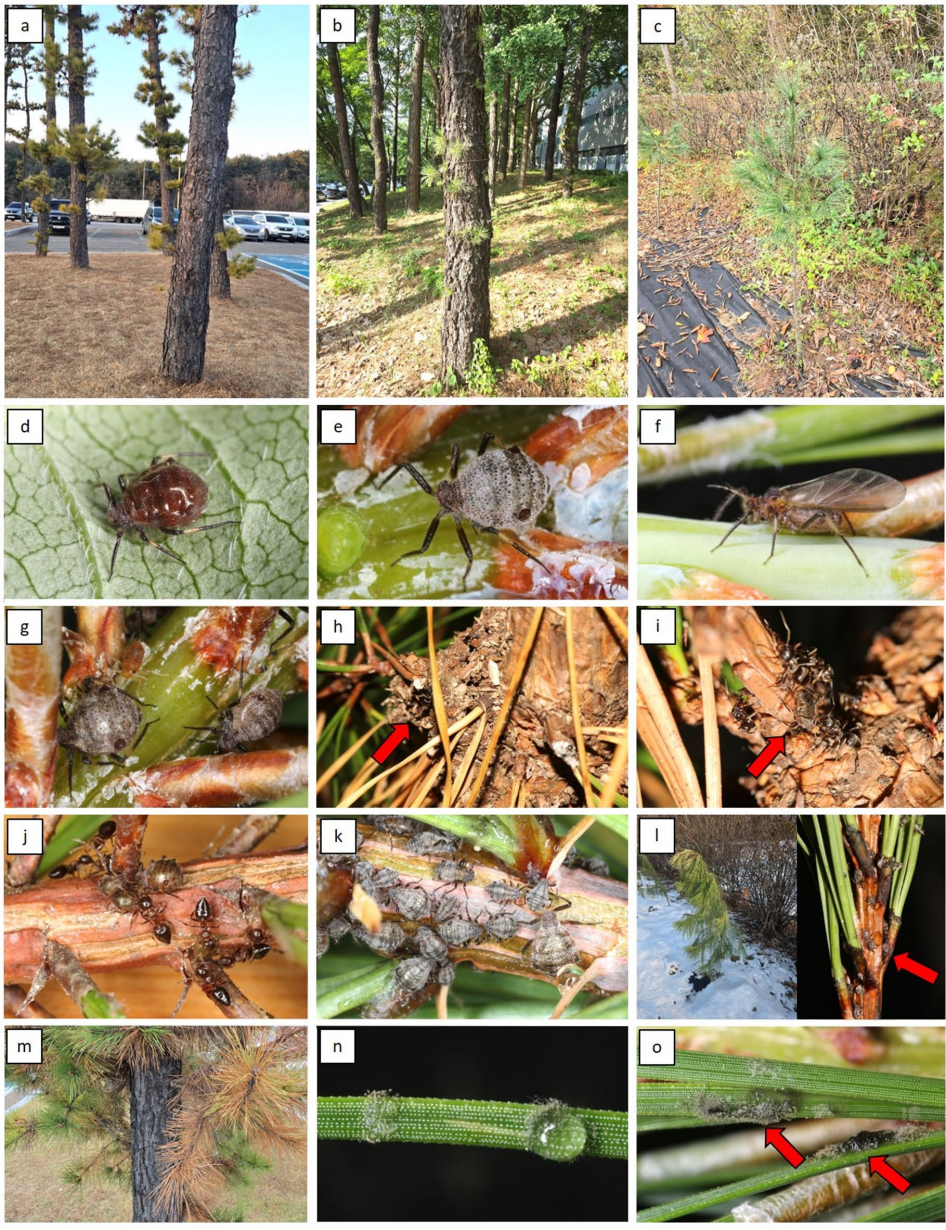
*Cinara watsoni*. In life: (**a, b**) *Pinus rigida*, (**c**) *P.* *taeda*, (**d**) apterous viviparous female, (**e**) apterous viviparous female (wax-covered form), (**f**) alate viviparous female, (**g**) colony on a young branch of *P. rigida *(in spring), (**h**) ants’ shelter constructed from soil and wood scraps, with colony under these shelter (in summer), (**i**) colony on a young branch of *P. taeda *(in fall), (**j**) colony on a young branch of *P. rigida *(in fall), (**k**) colony on a young branch of *P. rigida *(in winter, November), (**l**) colony on a young branch of *P. taeda* (in winter, January), (**m**) yellowing of the needles of *P.* *rigida*, (**n**) honeydew produced by aphids, (**o**) aphids causing black sooty mold on needles

Color. In life: head and thorax dark brown. ABD I brown with paired dark brown irregular dorsal patches. ABD II–VII brown. Whole body with slightly greyish-white wax (Fig. 1e). ANT I–II brown, ANT III–V light brown with 1/2 distal end brown, ANT VI brown. Fore, middle, and hind femora uniformly brown with light brown basal parts. Fore, middle and hind tibiae uniformly brown with light brown basal parts. Tarsi brown. SIPH dark brown (Fig. 1e). Pigmentation in mounted specimens: head and thorax dark brown. ABD I light brown with paired dark brown irregular dorsal patches. ABD II–VII brown (Fig. 3a). ANT I–II brown, ANT III–V light brown with 1/2 distal end brown, ANT VI brown (Fig. 3f). Fore, middle, and hind femora uniformly brown with light brown basal parts. Fore, middle and hind tibiae uniformly brown with light brown basal parts (Fig. 3g). Tarsi, SIPH, Cauda, and GP brown. Morphometric characters: body elongated oval (Fig. 3a). HW 0.49–0.53 × ANT. ANT 0.38–0.45 × BL. ANT III with 5–11 secondary rhinaria, shorter than ANT IV+V+VI. ANT IV shorter than ANT V with 1–3 secondary rhinarium. ANT V longer than ANT VI with one rounded primary rhinarium with one small rounded secondary rhinarium. ANT VI with PT 0.35–0.45 × BASE, with one rounded primary rhinarium and 4–5 accessory rhinaria. Other antennal ratios: VI/III 0.29–0.37, V/III 0.44–0.56, IV/III 0.35–0.50. LS ANT III 1.92–2.33 × BD III. Rostrum reaches ABD IV–V. URS 0.51–0.57 × ANT III, 1.48–1.81 × ANT VI and 0.92–1.00 × HT II with 6 fine accessory setae (Fig. 3c). HT I basal length 0.69–0.87 × dorsal, 0.34–0.43 × ventral. HT II 0.53–0.61 × ANT III and 1.56–1.81 × ANT VI. ABD VIII in form of broken band and few small scleroites with 12–16 setae. GP U-shaped with 43–58 fine setae (Fig. 3d). SIPH cone 9.71–12.95 × SIPH pore (Fig. 3e). TIBIAE III covered with long and fine setae on dorsal side longer (0.11–0.14 mm) than the width of TIBIAE III at midpoint (Fig. 3g). Cauda semi-circular with many fine, pointed setae. Fore wings with media branched twice (Fig. 3b).

### *Cinara watsoni* Tissot, 1939

#### Apterous viviparous female – re-description

**Fig. 5:**
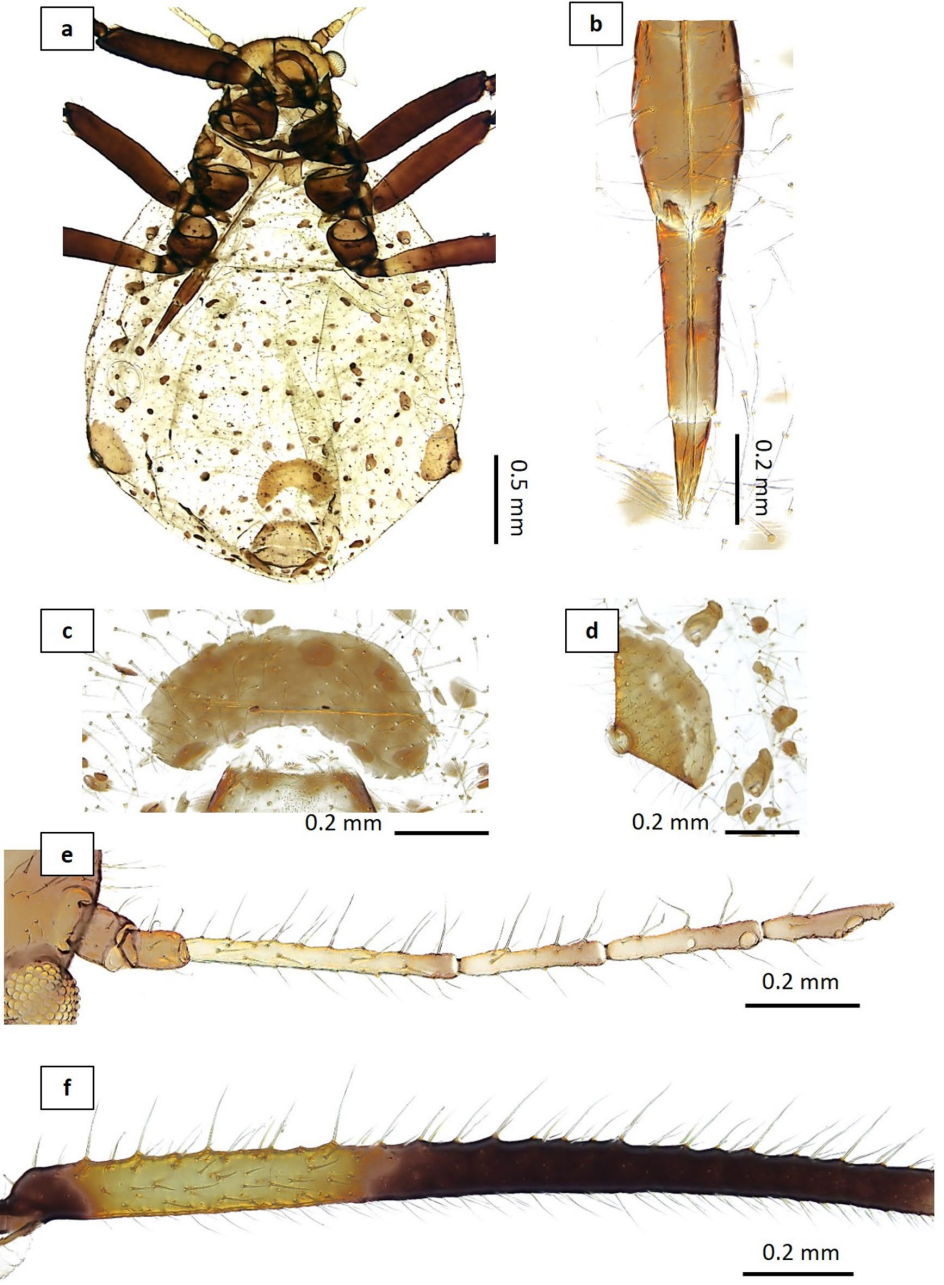
Morphological characters of *Cinara watsoni*, apterous viviparous female (LMHCWAP-7): (**a**) body, (**b**) URS, (**c**) GP (LMHCWAP-3), (**d**) SIPH, (**e**) ANT (LMHCWAP-2), (**f**) hind tibia

Figures 4d, e and 5; Table 2.

**Table 2:** Biometric measurement of *Cinara watsoni*. (min.–max.) mean length (mm). Measurements from the original description are shown for comparison with newly examined non-type specimens.

Species	*Cinara watsoni* (orig. descr. by Tissot, 1939)		*Cinara watsoni*	
Character	apterous viviparous female	alate viviparous female	apterous viviparous female (*n* = 34)	alate viviparous female (*n* = 8)
BL	3.50	3.40	(2.62–4.15) 3.54	(2.51–4.41) 3.18
MaxW	-	-	(1.79–3.28) 2.40	(1.19–2.44) 1.64
HW	(0.79–0.88) 0.85	(0.82–0.89) 0.85	(0.74–0.92) 0.81	(0.67–0.84) 0.73
ANT I	-	-	(0.09–0.13) 0.10	(0.07–0.10) 0.09
ANT II	-	-	(0.09–0.12) 0.10	(0.08–0.11) 0.09
ANT III	(0.47–0.53) 0.49	(0.45–0.50) 0.47	(0.43–0.67) 0.53	(0.38–0.57) 0.46
ANT IV	(0.28–0.30) 0.29	(0.24–0.30) 0.28	(0.20–0.33) 0.27	(0.16–0.30) 0.23
ANT V	(0.29–0.31) 0.30	(0.29–0.32) 0.30	(0.22–0.35) 0.28	(0.22–0.30) 0.26
BASE	(0.16–0.19) 0.17	(0.16–0.17) 0.16	(0.15–0.20) 0.17	(0.14–0.18) 0.16
PT	(0.05–0.07) 0.06	(0.04–0.05) 0.05	(0.04–0.06) 0.05	(0.04–0.05) 0.05
URS	-	-	(0.30–0.38) 0.32	(0.31–0.34) 0.32
FEMORA III	-	-	(1.05–1.72) 1.40	(0.96–1.58) 1.24
TIBIAE III	-	(2.1–2.4) 2.2	(1.55–2.56) 2.10	(1.49–2.52) 1.95
HT Ib	-	-	(0.04–0.06) 0.05	(0.04–0.05) 0.05
HT Id	-	-	(0.08–0.11) 0.10	(0.06–0.11) 0.08
HT Iv	-	-	(0.14–0.20) 0.17	(0.13–0.18) 0.15
HT II	-	-	(0.25–0.34) 0.30	(0.24–0.33) 0.29
SIPH cone	(0.38–0.47) 0.43	(0.37–0.42) 0.39	(0.33–0.48) 0.42	(0.26–0.44) 0.32
GP L	-	-	(0.24–0.36) 0.30	(0.17–0.31) 0.24
GP W	-	-	(0.42–0.68) 0.56	(0.37–0.58) 0.47
FWL	-	-	-	(3.17–4.70) 3.77

**Fig. 6:**
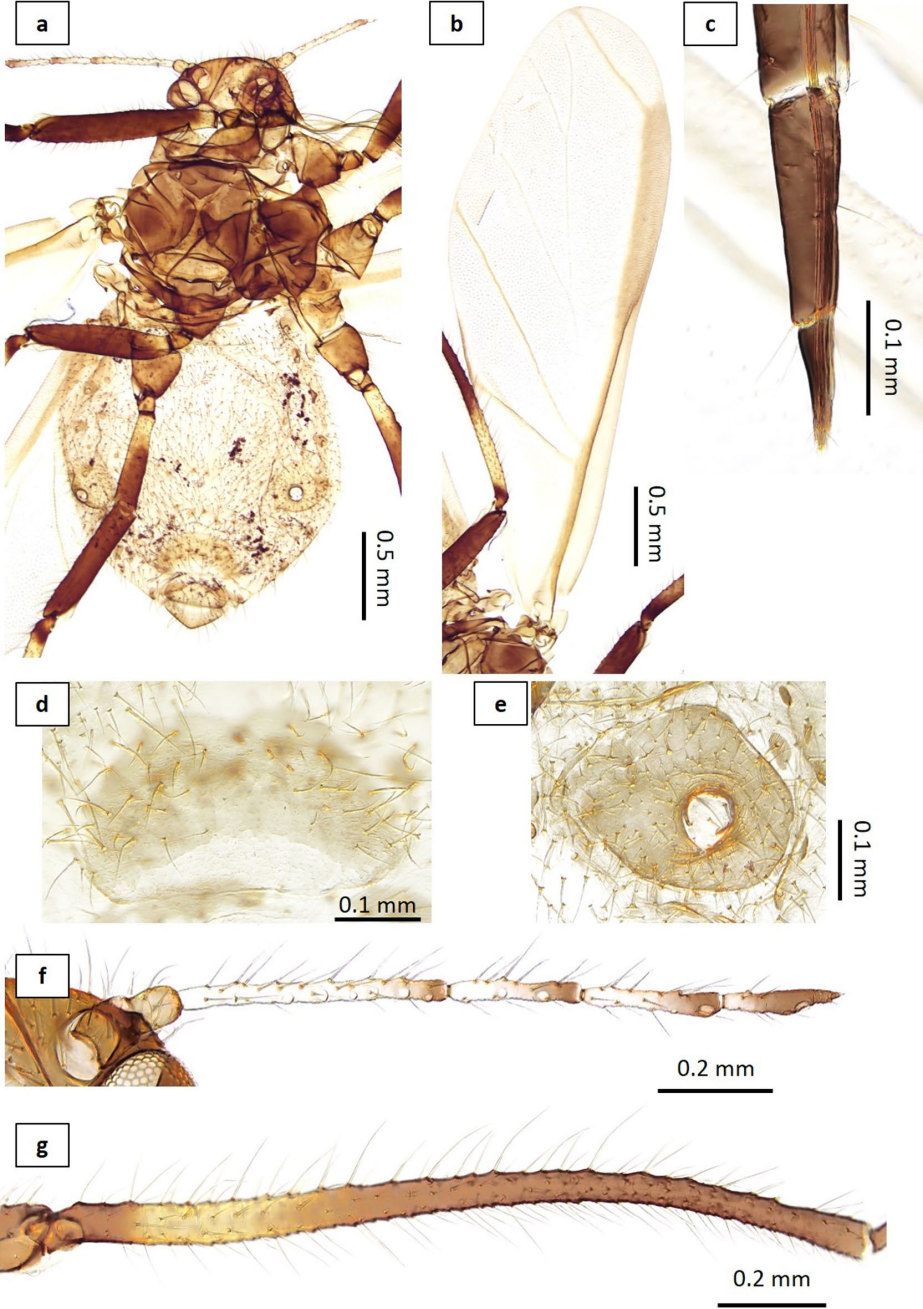
Morphological characters of *Cinara watsoni*, alate viviparous female (LMHCWAL-2): (**a**) body, (**b**) forewing, (**c**) URS, (**d**) GP (LMHCWAP-1), (**e**) SIPH (LMHCWAP-1), (**f**) ANT, (**g**) hind tibia (LMHCWAP-1)

Color. In life: head and thorax dark chestnut-brown (Fig. 4d) and abdomen brown with dark brown markings, whole body dusted with greyish-white wax (Fig. 4e). ANT I brown, ANT II–III light brown, ANT IV–V light brown with 1/3 distal end brown. ANT VI light brown with 2/3 distal end brown. Femora dusky brown. Fore tibiae uniformly dark, middle and hind tibiae with light brown basal parts. Tarsi, SIPH dark brown. Pigmentation in mounted specimens: head and thorax brown. Abdomen light brown (Fig. 5a). ANT I brown, ANT II–III light brown, ANT IV–V light brown with 1/3 distal end brown. ANT VI light brown with 2/3 distal end brown (Fig. 5e). Femora dusky brown. Fore tibiae uniformly dark, middle and hind tibiae uniformly dark with light brown basal parts (Fig. 5f). Tarsi, SIPH, Cauda, and GP brown. Morphometric characters: body elongated oval (Fig. 5a). HW 0.43–0.60 × ANT. ANT 0.36–0.50 × BL. ANT III with 0–1 secondary rhinaria, shorter than ANT IV + V+VI. ANT IV longer than ANT VI with 0–3 rounded secondary rhinaria. ANT V longer than ANT VI with one rounded primary rhinarium with 0–3 small rounded secondary rhinaria. ANT VI with PT 0.25–0.36 × BASE, with one rounded primary rhinarium and 5–6 accessory rhinaria. Other antennal ratios: VI/III 0.36–0.51, V/III 0.45–0.61, IV/III 0.43–0.57. LS ANT III 3.07–4.77 × BD III. Rostrum reaches ABD III–IV. URS 0.54–0.72 × ANT III, 1.28–1.70 × ANT VI and 0.90–1.24 × HT II with 4 fine accessory setae (Fig. 5b). HT I basal length 0.45–0.66 × dorsal, 0.25–0.35 × ventral. HT II 0.48–0.68 × ANT III and 1.25–1.59 × ANT VI. ABD VIII in form of broken band and few small scleroites with 13–19 setae. GP U-shaped with 26–38 fine setae (Fig. 5c). SIPH cone 5.17–7.72 × SIPH pore (Fig. 5d). TIBIAE III covered with long fine setae on dorsal side longer (0.14–0.20 mm) than the width of TIBIAE III at midpoint (Fig. 5f). Cauda semi-circular with many fine, pointed setae.

#### Alate viviparous female – re-description

Figures 4f and 6; Table 2.

**Fig. 7:**
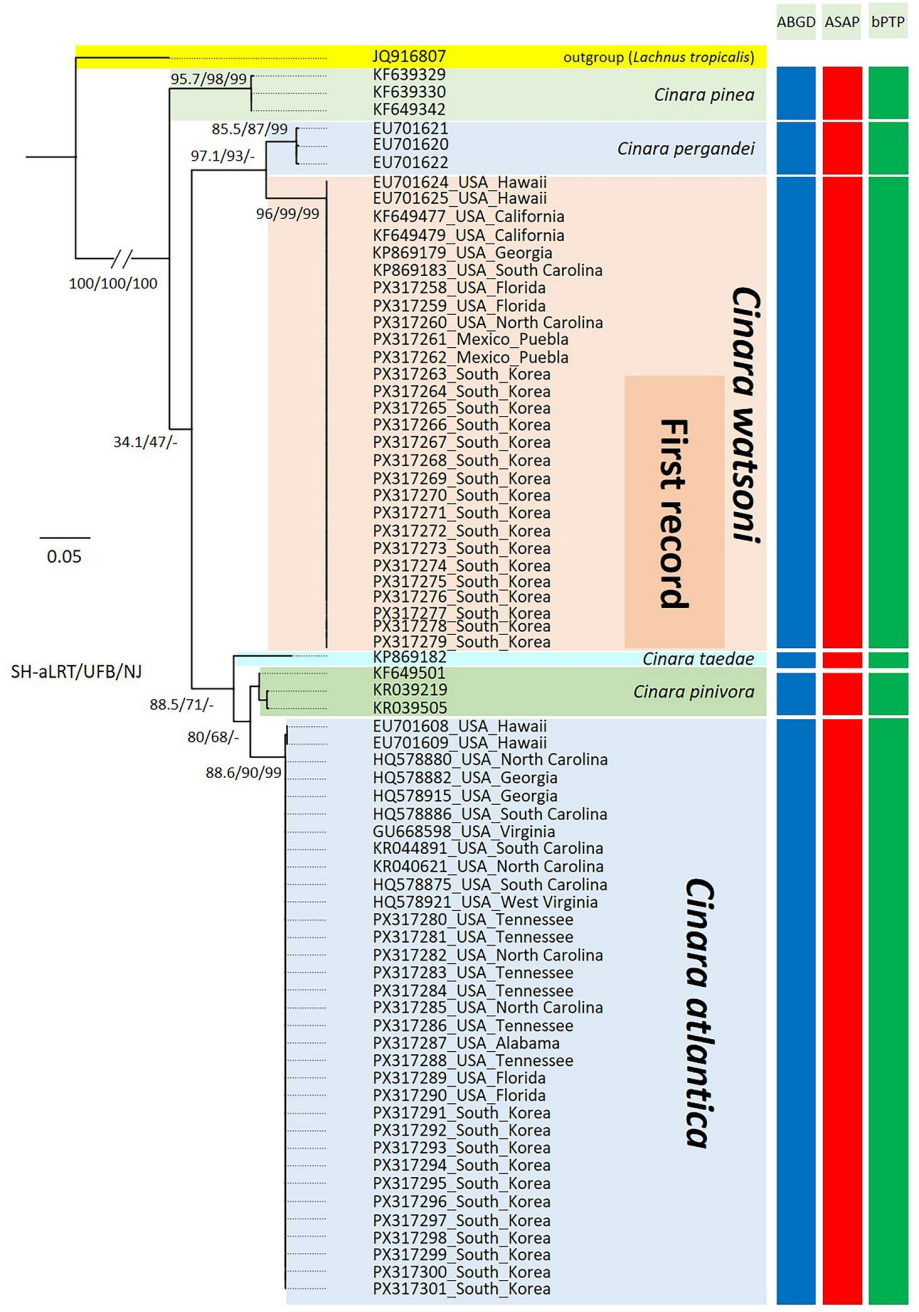
Phylogenetic analysis of *Cinara* species associated with *Pinus* *rigida* and *P*. *taeda*, including species delimitation results. Each node is annotated with two measures of branch support: Maximum Likelihood (ML) and Neighbor-Joining (NJ) values

Color. In life: head and thorax dark chestnut-brown and abdomen brown with dark brown markings, the whole body dusted with greyish-white wax (Fig. 4f). ANT I–II brown, ANT III light brown with 3/4 distal end brown, ANT IV–V light brown with 1/2 distal end brown. ANT VI light brown with 2/3 distal end brown. Fore, middle and hind femora dusky brown with light brown basal parts. Fore tibiae uniformly dark, middle and hind tibiae uniformly dark with pale basal parts. Tarsi dark brown, SIPH brown. Pigmentation in mounted specimens: head and thorax brown. Abdomen light brown (Fig. 6a). ANT I–II brown, ANT III light brown with 3/4 distal end brown, ANT IV–V light brown with 1/2 distal end brown. ANT VI light brown with 2/3 distal end brown (Fig. 6f). Fore, middle, and hind femora dusky brown with light brown basal parts. Fore tibiae uniformly dark, middle and hind tibiae uniformly dark with light brown basal parts (Fig. 6g). Tarsi, SIPH, Cauda, and GP brown. Morphometric characters: body elongated oval (Fig. 6a). HW 0.50–0.62 × ANT. ANT 0.36–0.46 × BL. ANT III with 3–8 secondary rhinaria, shorter than ANT IV + V+VI. ANT IV longer than ANT VI with 0–3 rounded secondary rhinaria. ANT V longer than ANT VI with one rounded primary rhinarium 1–3 small rounded secondary rhinarium. ANT VI with PT 0.27–0.33 × BASE, with one rounded primary rhinarium and 5–6 accessory rhinaria. Other antennal ratios: VI/III 0.36–0.51, V/III 0.49–0.67, IV/III 0.43–0.56. LS ANT III 2.97–4.11 × basal BD III. Rostrum reaches ABD III–IV. URS 0.57–0.85 × ANT III, 1.39–1.84 × ANT VI and 0.98–1.29 × HT II with 4 fine accessory setae (Fig. 6c). HT I basal length 0.46–0.68 × dorsal, 0.26–0.36 × ventral. HT II 0.56–0.69 × ANT III and 1.28–1.68 × ANT VI. ABD VIII in form of broken band and few small scleroites with 14–18 setae. GP U-shaped with 42–61 fine setae (Fig. 6d). SIPH cone 4.59–6.78 × SIPH pore (Fig. 6e). TIBIAE III covered with long and fine setae on dorsal side longer (0.17–0.21 mm) than the width of TIBIAE III at midpoint (Fig. 6g). Cauda semi-circular with many long fine, pointed setae. Fore wings with media branched twice (Fig. 6b).

### Molecular analyses

#### Genetic divergence

The intraspecific and interspecies genetic divergences (GDs) for six species (*Cinara atlantica*, *C*. *pergandei*, *C*. *pinea*, *C*. *pinivora*, C. *taedae*, and *C*. *watsoni*) were analyzed using COI sequences. The average intraspecific GD was 0.02%, with a maximum value of 0.90%. For *C*. *atlantica*, the intraspecific GD ranged from 0.00% to 0.10%, whereas for *C*. *watsoni* it was consistently 0.00%. The highest intraspecific GD was observed in *C*. *pinivora* (0.90%). The minimum interspecific GD among all six species was 3.10%, while the maximum interspecific divergence reached 10.00% (mean: 6.80%). All species exhibited a clear barcode gap, with the maximum intraspecific GD (0.90%) well separated from the minimum interspecific GD (3.10%).

#### Phylogenetic analyses and species delimitation

Maximum likelihood (ML) and Neighbour-joining analysis (NJ) inferred from the COI gene showed that multiple aphid samples were divided into six clades each corresponding to one species (Fig. 7). The species delimitation methods of ABGD, ASAP, and bPTP yielded six molecular operational taxonomic units (MOTUs), respectively. The pairwise distance gap approach (ABGD) with default settings (X = 0.5) identified six species with a barcode gap of 3.50%. The first ASAP partition (score = 1.5) provided the best-fit, delimiting six species at a threshold distance of 2.03% (K80). Similarly, the bPTP analysis supported the presence of six species with high posterior probabilities. The clear separation of clades confirms that the COI barcode region is an effective marker for distinguishing closely related *Cinara* species. Molecular analyses of the COI gene from *C. atlantica* and *C*. *watsoni* collected across various countries provide strong support for the identification of the *Cinara* species found in South Korea as *C. atlantica* and *C*. *watsoni*. Notably, the population of *C*. *atlantica* discovered on *Pinus densiflora* in South Korea was unequivocally identified as *C*. *atlantica*, confirming its recent host range expansion from non-native (*P*. *banksiana*, *P*. *rigida*) to native pines.

**Fig. 8:**
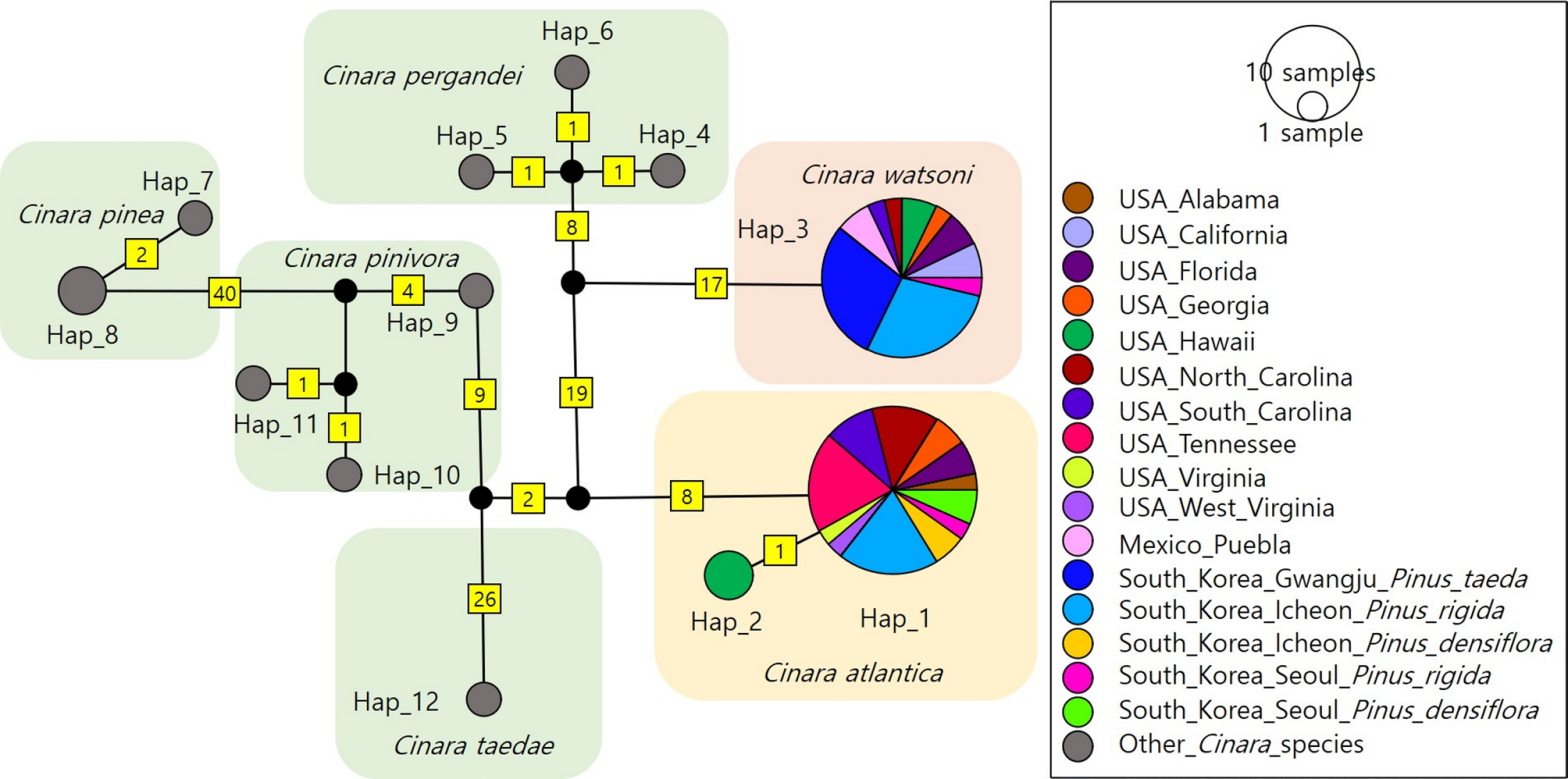
TCS network of nine haplotypes of the 71 COI sequences. The pi size is proportional to the haplotype frequency. The number in the box indicates the number of mutations.

#### Haplotype analysis

The TCS network analysis detected 12 haplotypes from the 71 ingroup COI sequences (Fig. 8, Fig. S1, Fig. S2). Details of haplotype distribution are provided in Supplementary Table 1. For *C*. *atlantica*, 35 sequences were grouped into two haplotypes (Hap_1 and Hap_2). Haplotype 1 (Hap_1) was identified in specimens collected from South Korea (Seoul, Icheon) and the United States (Alabama, Florida, Georgia, North Carolina, South Carolina, Tennessee, Virginia, and West Virginia). Haplotype 2 (Hap_2) occurred exclusively in specimens from Hawaii, USA. Notably, Hap_1 comprised specimens collected from *P*. *densiflora* in South Korea as well as those associated with non-native pines, indicating a shared haplotype across both native and introduced host species. For *C*. *watsoni*, all 28 sequences were of the same haplotype (Hap_3).

#### Biology

In this study, the following biological observations refer to *C. atlantica* detected on *Pinus rigida* in Icheon, and to *C. watsoni* detected on *P. rigida* in Icheon and on *P. taeda* in Gwangju. In South Korea, *C. atlantica* exhibits an anholocyclic life cycle, characterized by the continuous presence of both apterous and alate viviparous females throughout the year and an absence of sexuales. Colonies exhibited pronounced seasonal fluctuations in colony size and distribution on host plants. *C*. *atlantica* was first documented in South Korea^31^, who reported its occurrence on newly flushed shoots and young branches of non-native pines (*P*. *rigida* and *P*. *banksiana*) during the spring months (April–June). Since that initial record, no further ecological studies had been undertaken until the now. In this study, colonies were additionally detected on young branches of the native pine (*P*. *densiflora*) (Figs. 1b, c, j). These colonies were composed exclusively of apterous and alate viviparous females, with no evidence of sexual morphs.

Field monitoring of *C*. *atlantica* on *P*. *rigida* revealed a distinct seasonal pattern in colony dynamics. In spring (mid-April to early-June), apterous and alate viviparous females were observed feeding on newly flushed shoots, forming small colonies of approximately 10 individuals at the shoot tips (Fig. 1f). By early-June, colony size increased to around 20 individuals, primarily distributed along young branches. During this time, alates dispersed to adjacent *P*. *rigida*, where they established new colonies. In summer (late-July to August), population density declined sharply, with only 2–3 individuals scattered along branches, indicating a marked seasonal reduction. In fall (mid-September to October), densities increased again, to about 20 individuals at branch bases (Fig. 1g). Numerous alates were present during this period and in October, they dispersed to nearby *P*. *rigida*, initiating new small colonies. In early-winter (November to December), colonies persisted with approximately 20 individuals composed of both apterous and alate viviparous females (Fig. 1h, i). However, in mid-winter (January), colony size declined rapidly to fewer than 10 individuals, with some nymphs migrating toward the roots. In February, only 1–2 nymphs remained on the branches, and no aphids were detected on aboveground parts in March. In early April, nymphs were again detected on newly developing aboveground shoots.

Observations on *Pinus rigida* and *Pinus taeda* revealed that *Cinara watsoni* exhibits an anholocyclic life cycle with seasonal fluctuations in colony size and distribution. In spring (late-April to May), small colonies consisting of apterous and alate viviparous females were observed feeding on newly emerging shoots (Fig. 4g). By late May, each colony on the shoot tips contained approximately 10 individuals, and by mid-June, colony size increased to 20 individuals, primarily distributed along young branches. During this period, alates dispersed to nearby *P*. *rigida* and *P*. *taeda*, successfully establishing new colonies. In summer (early-July to late-August), population density decreased sharply. Only 1–2 individuals were found on *P*. *rigida* branches, with solitary individuals occasionally observed beneath the trunk bark. On *P*. *taeda*, 2–3 individuals were detected inside shelters constructed by attending ants (Formicidae: *Lasius* sp.) (Figs. 4h, i), while no aphids were observed on exposed branches. In fall (mid-September to October), population density increased again, colonies of approximately 20 individuals forming on branches. Colonies on *P*. *rigida* were frequently attended by ants (Formicidae: *Crematogaster* sp.) (Fig. 4j). A few alate nymphs were also observed during this period. In early winter (November–December), colonies persisted with approximately 20 individuals composed of both apterous and alate viviparous females (Fig. 4k). However, in mid-winter (January), colony size on branches declined rapidly to fewer than 10 individuals (Fig. 4l), and some nymphs migrated toward the roots or into crevices beneath the trunk bark. By mid-February, only 1–2 nymphs remained on the branches, and no aphids were observed on aboveground parts in March. In April, nymphs were again detected on newly developing aboveground shoots. No parasitoids were detected for *C*. *atlantica* and *C*. *watsoni*. Predatory lady beetles, however, were observed near the aphid colonies in June.

#### Damage

For both species, increased colony density during June and again in October–November led to the development of sooty mold on leaves and branches, caused by honeydew excreted by the aphids (Figs. 1k, l and 4n, o). Notably, branches heavily infested with *C. atlantica* and *C*. *watsoni* showed pronounced yellowing of needles (Figs. 1j and 4m).

## Discussion

### Life cycle plasticity as a key factor in invasion success

Field observations suggest that *Cinara atlantica* and *C*. *watsoni* predominantly reproduce anholocyclically under South Korean climatic conditions, as no sexual morphs or overwintering eggs were detected throughout the surveys. In contrast, reports from their native range indicate geographic variability in life cycle patterns. Oviparous females and males of *C*. *atlantica* have been observed in October, and those of *C*. *watsoni* in October and November^39^, suggesting that holocycly may occur under certain climatic conditions. More recent compilations also document anholocycly in *C*. *atlantica*^16^, whereas direct evidence for anholocycly in *C. watsoni* remains limited^36,47^. The absence of sexual morphs in South Korea therefore likely reflects local ecological or climatic adaptation rather than a strictly fixed life-history strategy.

Notably, the regions represented in our haplotype analyses, including several U.S. states and Mexico, experience relatively mild winters, conditions under which *Cinara* species are typically anholocyclic. This climatic context suggests that the introduced populations in South Korea likely originated from predominantly anholocyclic source populations rather than from holocyclic populations inhabiting colder regions.

Such a strategy may parallel that of *Cinara tujafilina* (Del Guercio, 1909), which exploits thermally stable microhabitats to survive winter and maintain reproduction across generations^46^. It is therefore plausible that *C. atlantica* and *C. watsoni* similarly overwinter in protected sites such as beneath bark or within soil layers. Although this hypothesis remains speculative, it highlights the importance of targeted ecological studies to clarify overwintering mechanisms.

If invasive populations were derived mainly from clonal, anholocyclic sources, founder effects associated with parthenogenetic reproduction may have further reinforced life cycle uniformity in the introduced range. Such processes could limit life-history variability while facilitating rapid population establishment under favorable conditions. Taken together, life cycle plasticity combined with behavioral adaptation to microclimatic refugia appears to be a key trait promoting the establishment and spread of invasive aphids in novel environments^46^.

Previous studies from the native range documented pronounced seasonal fluctuations in population density, with peaks typically occurring during winter or early spring, but did not examine morph composition or overwintering stages^47–49^. In contrast, our continuous monitoring revealed the presence of apterous viviparous females and nymphs during early winter and only nymphs thereafter, with no sexual morphs or eggs detected. These findings support an anholocyclic overwintering strategy, at least in part of the species’ range.

Nevertheless, the extent to which adults reproduce during winter and the precise dynamics of overwintering remain unresolved under field conditions alone. Controlled laboratory experiments simulating winter temperature regimes are therefore required to validate these interpretations. Furthermore, historical reports of sexual morphs should be re-evaluated in light of potential climatic effects and possible misidentification^39^.

### Host range expansion and ecological implications

A significant finding of this study is the expansion of *C*. *atlantica* onto the native pine (*P*. *densiflora*) in South Korea. This broadening of host range indicates its potential to integrate into native forest ecosystems, a pattern previously associated with rapid population growth and heightened ecological impacts in other *Cinara* invasions^26,30^. In contrast, *C*. *watsoni* was observed only on imported pines (*P*. *rigida* and *P*. *taeda*). However, its persistent colonies, mutualistic interactions with ants, and high population densities suggest potential to expand onto native pines in the future.

Comparable risks have been reported in Hawaii, where a three-year survey documented invasive aphids feeding on 64 native plants, including many endangered endemics, illustrating how host shifts can threaten biodiversity and facilitate virus transmission^50^. These findings are analogous to the situation in South Korea, underscoring the urgent need for monitoring, quarantine measures, and proactive management to mitigate the ecological risks posed by *C*. *atlantica* and *C*. *watsoni*.

### Molecular evidence and invasion pathways

This study combined genetic divergence, phylogenetic analyses, species delimitation, and haplotype network data to clarify the identity and invasion routes of *C*. *atlantica* and *C*. *watsoni* in South Korea. COI analysis revealed a clear barcode gap (0.90% intraspecific vs. 3.10% interspecific), confirming distinct species boundaries. *C*. *watsoni* showed no intraspecific divergence, while *C*. *atlantica* displayed minimal variation (0.10%), supporting the reliability of COI for species identification. Phylogenetic trees (ML, NJ) and species delimitation methods (ABGD, ASAP, bPTP) consistently identified six well-supported clades and molecular operational taxonomic units (MOTUs), validating the distinctiveness of the analyzed species.

The haplotype network provided additional insight into invasion dynamics. *C*. *atlantica* exhibited two haplotypes, Hap_1 and Hap_2. Hap_1 was widely distributed, occurring in both the United States and South Korea, and was detected in colonies collected from both native *Pinus densiflora* and non-native pines in South Korea. This pattern indicates that South Korean populations share a common haplotype with populations sampled in North America, and that host range expansion to *P*. *densiflora* occurred after establishment in South Korea. However, because Hap_1 may be widely distributed across multiple regions, these data do not allow definitive identification of the precise geographic source population. Hap_2 was restricted to Hawaii, further supporting the likelihood that the Korean invasion stems from the more widespread Hap_1 lineage. *Cinara* *watsoni* showed a single haplotype, Hap_3, across all sampled regions, including South Korea, the United States, and Mexico. The absence of genetic diversity and the widespread distribution of Hap_3 imply that this species was introduced to South Korea from North America through a single or few introduction events, likely facilitated by international trade in ornamental and forestry pines. Subsequent clonal reproduction via obligate parthenogenesis likely promoted its establishment.

Collectively, these findings indicate that *C*. *atlantica* and *C*. *watsoni* populations in South Korea are genetically uniform lineages that most likely originated from North American source populations. The presence of identical haplotypes in both ranges supports a scenario of recent invasion via anthropogenic pathways, such as the movement of live pine plants or timber products. The ability of *C*. *atlantica* to expand its host range to native *P*. *densiflora* further underscores its ecological threat to Korean forest ecosystems. Therefore, the evidence strongly supports that both species have invaded South Korea from North America, and their life history traits, particularly anholocycly and life cycle plasticity, have likely facilitated their establishment and spread in this new environment. Future genomic studies and trade pathway analyses are warranted to further validate the invasion routes and assess potential management strategies.

### Implications for international pest management

The host range expansion of *C*. *atlantica* to the native pine (*P*. *densiflora*) and the establishment of *C*. *watsoni* on introduced pines highlight the ecological risks these invasive aphids pose to South Korea’s coniferous forests. Colonization of *P*. *densiflora* by *C*. *atlantica* represents a potential long-term threat to the stability of natural pine forests, including protected areas such as national parks. Likewise, the persistent presence of *C*. *watsoni* on non-native pines suggests that its range may further expand in the absence of effective management. Year-round parthenogenesis, mutualistic interactions with ants, and the lack of natural enemies in invaded regions facilitate the invasion success of these species. The low genetic divergence between populations in South Korea and those sampled elsewhere is consistent with recent introductions followed by rapid establishment. These findings highlight the importance of sustained monitoring of both known hosts and adjacent native vegetation that may serve as future reservoirs. Surveillance should be prioritized in urban green spaces and national parks, where early detection of emerging populations is essential for timely intervention. Understanding their life cycles in the introduced range will also help predict seasonal dynamics and identify vulnerable stages for control.

From a broader global perspective, management and monitoring of alien *Cinara* species are equally critical, as their host plants are widely cultivated beyond their native ranges, particularly in parks, plantations, and urban greenery^51^. In Europe, invasive *Cinara* such as *C*. *cedri* Mimeur, 1936 and *C*. *laportei* (Remaudière, 1954) (both of African origin) and *C*. *curvipes* (Patch, 1912) (North American origin) have been the focus of extensive research, with occurrences documented across Bulgaria, China, Cyprus, Slovenia, Japan, Korea, Poland, and Türkiye^52–59^. In Türkiye, natural enemy surveys of *C*. *cedri* revealed 28 associated species, indicating potential avenues for biocontrol^60^. More recently, the North American bark aphid *C*. *splendens* (Gillette & Palmer, 1924) was detected on *Pseudotsuga menziesii* in the Czech Republic^61^. Considering the widespread planting of Douglas fir in European parks and gardens, its further spread seems inevitable and may pose risks comparable to cedar aphids in southern France^62,63^. Collectively, these cases demonstrate that *Cinara* invasions represent not only a local but also a global management challenge. Strengthened phytosanitary measures, international cooperation, and continuous monitoring are therefore urgently required to mitigate their ecological and economic impacts.

## Materials and methods

### Field survey and identification

*Cinara atlantica* was collected at two locations in South Korea. On 16 December 2020, a colony of *C*. *atlantica* was discovered on *P*. *rigida* in Seoul, and additional specimens were collected from a street-planted native pine (*P*. *densiflora*) located approximately 15 m away (Figs. 1b, c). Furthermore, On 7 November 2022, *C*. *atlantica* was detected on *P*. *rigida* in Icheon, Gyeonggi Province (Fig. 1a )Specimens were also collected from an ornamental *P*. *densiflora* planted approximately 20 m away. All individuals collected from *P*. *densiflora* were preserved for precise species identification, which prevented further field monitoring of these colonies. The population at the Icheon site was subsequently monitored monthly from 7 November 2022 to 2 May 2025.

*Cinara watsoni* was collected at three locations in South Korea. The species was first collected on 7 November 2022 from *P*. *rigida* planted in Icheon (Fig. 4a)where monthly monitoring was conducted from the date of detection until 2 May 2025. In Seoul, only a few individuals were detected on 15 October 2023, and because all specimens were collected for identification, no further monitoring was performed at this site. Additionally, a population was found on *P*. *taeda* (Fig. 4b) in Gwangju on 19 June 2024 and was monitored monthly until 2 May 2025. Comparative specimens of both *Cinara* species were collected at several locations in USA and Mexico.

The aphid samples were preserved in 90% ethanol, and slide-mounted specimens were prepared in Canada balsam following the method described by^64^. Measurements and digital images were obtained using Leica DMC 5400 (Leica Z16 APO) and Leica DM 4000B (Active Measure version 3.0.3; Mitani Co. Ltd., Japan) cameras system. The abbreviations used in the descriptions follow those proposed previously^65^. ANT: antennae; ANT I, ANT II, ANT III, ANT IV, ANT V, BASE, and PT: antennal segments I, II, III, IV, V, base of VI, and processus terminalis of antennomere VI respectively; BD III: basal articular diameter of ANT III; LS ANT III: length of the longest setae of ANT III; BL: body length; MaxW: greatest body width; HW: greatest head width across compound eyes; GP: genital plate; HT I: first segment of hind tarsus; HT Ib: basal length of HT I; HT Id: dorsal length of HT I; HT Iv: ventral length of HT I; HT II: second segment of hind tarsus; URS: ultimate rostral segment (segment IV + V); ABD I–VIII, Abdominal tergites I–VIII, respectively; FEMORA III: hind femora; TIBIAE III: hind tibiae.

### Material examined

Type material was not directly examined in the present study. Instead, the redescriptions were prepared based on (i) detailed measurements and diagnostic characters reported in the original descriptions, and (ii) newly examined non-type specimens from South Korea and specimens collected from the native ranges of both species. To ensure comparability and taxonomic transparency, measurements from the original descriptions are explicitly provided alongside those of our material (Tables 1 and 2). Characters derived from the original type series are clearly distinguished from measurements obtained from newly collected specimens.

Minor discrepancies between measurements reported in the original descriptions and those obtained from the present material most likely represent normal intraspecific variation, geographic differentiation among populations, and methodological differences among studies, such as slide preparation and sampling effort. Nevertheless, all examined specimens conform to the diagnostic characters and overall morphology provided in the original descriptions, supporting their assignment to *Cinara atlantica* and *C. watsoni*.

Apterous viviparous female of *Cinara atlantica*: 2, South Korea, Seoul, Gwanak-gu, Sillim-dong (GPS: 37.4599, 126.9489), on *P*. *rigida*, 16.xii.2020, leg. M. Lee, [201216-LMH-2] (SNU); 3, South Korea, Seoul, Gwanak-gu, Sillim-dong (GPS: 37.4601, 126.9487), on *P*. *densiflora*, 16.xii.2020, leg. M. Lee, [201216-LMH-3] (SNU); 1, South Korea, Gyeonggi-do, Icheon-si, Majang-myeon (GPS: 37.2422, 127.3894), on *P*. *rigida*, 7.xi.2022, leg. M. Lee, [221107-LMH-3] (SNU); 2, ditto as Icheon-si (GPS: 37.2410, 127.3893), on *P*. *densiflora*, 7.xi.2022, leg. M. Lee, [221107-LMH-4] (SNU); 1, ditto as Icheon-si, on *P*. *rigida*, 22.xii.2022, leg. M. Lee, [221222-LMH-2] (SNU); 1, ditto as Icheon-si, on *P*. *rigida*, 12.i.2023, leg. M. Lee, [230112-LMH-1] (SNU); 1, ditto as Icheon-si, on *P*. *rigida*, 16.iv.2023, leg. M. Lee, [230416-LMH-1] (SNU); 1, ditto as Icheon-si, on *P*. *rigida*, 18.v.2023, leg. M. Lee, [230518-LMH-5] (SNU); 1, ditto as Icheon-si, on *P*. *rigida*, 13.vi.2023, leg. M. Lee, [230613-LMH-2] (SNU); 2, ditto as Icheon-si, on *P*. *rigida*, 23.ix.2023, leg. M. Lee, [230923-LMH-6] (SNU); 2, ditto as Icheon-si, on *P*. *rigida*, 15.x.2023, leg. M. Lee, [231015-LMH-1] (SNU); 1, ditto as Icheon-si, on *P*. *rigida*, 21.xi.2023, leg. M. Lee, [231121-LMH-3] (SNU); 1, ditto as Icheon-si, on *P*. *rigida*, 16.xii.2023, leg. M. Lee, [231216-LMH-1] (SNU); 1, ditto as Icheon-si, on *P*. *rigida*, 22.v.2024, leg. M. Lee, [240522-LMH-3] (SNU); 2, USA, Tennessee, Blount Co. (GPS: 35.6210, −83.8023), on *Pinus strobus*, 12.v.2004, leg. C. Favret (SNU); 2, USA, Tennessee, Blount Co. (GPS: 35.5633, −83.7687), on *Pinus virginiana*, 16.v.2004, leg. C. Favret (SNU); 1, USA, Tennessee, Blount Co. (GPS: 35.5633, −83.7687), on *Pinus strobus*, 16.v.2004, leg. C. Favret (SNU); 1, USA, Alabama, Calhoun (GPS: 33.6149, −85.7221), on *Pinus taeda*, 1.v.2009, leg. C. Favret (SNU); 1, USA, North Carolina, Wayne Co., Cliffs of the Neuse State Park, 5.vi.1958, leg. Brown (FSCA).

Alate viviparous female of *Cinara atlantica*: 1, ditto as Icheon-si, on *P*. *rigida*, 7.xi.2022, leg. M. Lee, [221107-LMH-3] (SNU); 3, ditto as Icheon-si, on *P*. *rigida*, 15.x.2023, leg. M. Lee, [231015-LMH-1] (SNU); 1, USA, North Carolina, Yancey Co., Mt. Mitchell State Park, 12.v.1970, leg G.F. Fedde (FSCA).

Apterous viviparous female of *Cinara watsoni*: 5, South Korea, Gyeonggi-do, Icheon-si, Majang-myeon (GPS: 37.2419, 127.3891), on *P*. *rigida*, 7.xi.2022, leg. M. Lee, [221107-LMH-3] (SNU); 6, ditto as Icheon-si, on *P*. *rigida*, 10.xii.2022, leg. M. Lee, [221210-LMH-1] (SNU); 1, ditto as Icheon-si, on *P*. *rigida*, 22.iv.2023, leg. M. Lee, [230422-LMH-1] (SNU); 1, ditto as Icheon-si, on *P*. *rigida*, 1.v.2023, leg. M. Lee, [230501-LMH-1] (SNU); 1, ditto as Icheon-si, on *P*. *rigida*, 13.vi.2023, leg. M. Lee, [230613-LMH-1] (SNU); 2, ditto as Icheon-si, on *P*. *rigida*, 23.ix.2023, leg. M. Lee, [230923-LMH-5] (SNU); 1, ditto as Icheon-si, on *P*. *rigida*, 15.x.2023, leg. M. Lee, [231015-LMH-2] (SNU); 1, South Korea, Seoul, Gwanak-gu, Sillim-dong (GPS: 37.4601, 126.9491), on *P*. *rigida*, 15.x.2023, leg. M. Lee, [231015-LMH-4] (SNU); 3, South Korea, Gyeonggi-do, Gwangju-si, Docheok-myeon (GPS: 37.3119, 127.3114), on *P*. *taeda*, 19.vi.2024, leg. M. Lee, [240619-LMH-1] (SNU); 1, ditto as Gwangju-si, on *P*. *taeda*, 22.ix.2024, leg. M. Lee, [240922-LMH-1] (SNU); 1, ditto as Gwangju-si, on *P*. *taeda*, 13.x.2024, leg. M. Lee, [241013-LMH-4] (SNU); 1, ditto as Icheon-si, on *P*. *rigida*, 1.xi.2024, leg. M. Lee, [241101-LMH-11] (SNU); 1, ditto as Gwangju-si, on *P*. *taeda*, 14.xii.2024, leg. M. Lee, [241214-LMH-1] (SNU); 1, ditto as Icheon-si, on *P*. *rigida*, 14.xii.2024, leg. M. Lee, [241214-LMH-2] (SNU); 1, ditto as Gwangju-si, on *P*. *taeda*, 12.i.2025, leg. M. Lee, [250112-LMH-1] (SNU); 2, ditto as Icheon-si, on *P*. *rigida*, 2.v.2025, leg. M. Lee, [250502-LMH-2] (SNU); 1, USA, North Carolina, Swain (GPS: 35.4566, −83.5057), on *P*. *rigida*, 13.vi.2006, leg. C. Favret (SNU); 2, Mexico, Puebla (GPS: 19.3497, −98.6202), on *Pinus* sp., 3.vi.2008, leg. C. Favret (SNU); 2, USA, Pennsylvania, Centre Co., State College, 10.ix.1964, leg J.O.P & A.N.T (FSCA).

Alate viviparous female of *Cinara watsoni*: 1, ditto as Icheon-si, on *P*. *rigida*, 10.xii.2022, leg. M. Lee, [221210-LMH-1] (SNU); 2, ditto as Icheon-si, on *P*. *rigida*, 13.vi.2023, leg. M. Lee, [230613-LMH-1] (SNU); 3, ditto as Gwangju-si, on *P*. *taeda*, 12.i.2025, leg. M. Lee, [250112-LMH-1] (SNU); 1, ditto as Icheon-si, on *P*. *rigida*, 2.v.2025, leg. M. Lee, [250502-LMH-2] (SNU); 1, USA, Pennsylvania, Centre Co., State College, 15.viii.1964, leg J.O.P & A.N.T (FSCA).

Depositories of slide-mounted specimens of *C*. *atlantica* and *C*. *watsoni* examined:

SNU—College of Agriculture and Life Sciences, Seoul National University, Seoul, Korea.

FSCA—Florida State Collection of Arthropods.

### Molecular analyses

#### Molecular protocol

Genomic DNA was extracted from individual samples collected from each colony using the DNeasy Blood & Tissue kit (Qiagen, Düsseldorf, Germany) following modified manufacturer protocols. We used a non-destructive method to confirm the morphological features. A 658 bp of the cytochrome oxidase I gene (COI) was amplified using the following primer pair: LepF 5′- ATTCAACCAATCATAAAGATATTGG-3′ and LepR 5′ TAAACTTCTGGATGTCCAAAAAATCA-3′^66^. Polymerase chain reaction (PCR) was performed using AccuPower PCR Premix (Bioneer, Daejeon, Republic of Korea) in 20 µl reaction volumes. The amplification protocol consisted of an initial denaturation at 94 °C for 3 min, followed by 35 cycles at 94 °C for 30 s, an annealing temperature of 45.2 °C for 30 s, extension at 72 °C for 1 min, and the final extension step at 72 °C for 5 min. PCR products were checked using 1.5% agarose gel electrophoresis, and sequenced at Bionics, Inc. (Seoul, Republic of Korea).

#### Sequence analysis and genetic divergence

In this study, a total of 72 COI sequences were analyzed. Since *C*. *atlantica* and *C*. *watsoni* have been reported in South Korea on non-native pine species (*P*. *rigida* and *P*. *taeda*), these two species were included, along with additional *Cinara* species associated with the same host plants, as selected from Favret C & Aphid Taxon Community^16^. Furthermore, 28 COI sequences of six species (*C*. *atlantica*, *C*. *pergandei* (Wilson, 1919), *C*. *pinea* (Mordvilko, 1895), *C*. *pinivora* (Wilson, 919), *C*. *taedae* Tissot, 1932, and *C*. *watsoni*) were retrieved from GenBank. The dataset also incorporated 44 novel COI sequences generated from specimens collected in the native range (United States and Mexico) and from South Korea. *Lachnus tropicalis* (van der Goot, 1916) (GenBank accession number: JQ916807) was used as an outgroup. All newly generated sequences were deposited in GenBank (accession numbers PX317258 to PX317301) and are detailed in Supplementary Table 1. Raw sequences were assembled and edited using SeqMan Pro version 7.1.0 (DNASTAR, Inc., Madison, WI, USA) and aligned in MEGA7^67^. Pairwise genetic distances were calculated under the Kimura 2-parameter (K2P) model with 1,000 bootstrap replicates to assess intra- and interspecific divergence^46^.

#### Phylogenetic analysis and species delimitation

Phylogenetic analyses were conducted using Maximum likelihood (ML) and Neighbor-joining analysis (NJ) methods. ML analyses were performed in IQ-TREE v2.4.7^68^ with 1,000 replicates of ultrafast bootstrap approximation (UFBoot) and 1,000 replicates of the SH-like approximate likelihood ratio test (SH-aLRT), under the best partition scheme and best-fit substitution models identified by PARTITION-FINDER2^69^. Result of analysis were visualized using FIGTREE v.1.4.4^70^. The NJ analysis was conducted using MEGA 7, which is based on the Kimura-2-parameter (K2P) model^67^. We followed three DNA-based species delimitation methods proposed by Wieczorek and Sawka^71^: Automatic Barcode Gap Discovery (ABGD)^72^, Assemble Species by Automatic Partitioning (ASAP)^73^, and Bayesian implementation of the Poisson Tree Processes model (bPTP)^74^.

#### Population genetic analyses

Variable and parsimony-informative sites, as well as the number of haplotypes (h), were estimated using DnaSP v6.12.03^75^. The haplotype list generated in DnaSP was used to identify unique haplotypes and to determine the number of private haplotypes for each population (Supplementary Table 1). Haplotype relationships were then visualized by constructing a statistical parsimony network using the TCS method^76^ as implemented in PopART^77^.

## Supplementary Information

Below is the link to the electronic supplementary material.

Supplementary Material 1

## Data Availability

The datasets generated and/or analysed during the present study are available in the GenBank repository under accession numbers PX317258 to PX317301. All sequence data are publicly accessible.

## References

[1] Pimentel, D., Zuniga, R. & Morrison, D. Update on the environmental and economic costs associated with alien-invasive species in the United States. *Ecol. Econ.* **52**, 273–288. 10.1016/j.ecolecon.2004.10.002 (2005).

[2] Hulme, P. E. Trade, transport and trouble: managing invasive species pathways in an era of globalization. *J. Appl. Ecol.* **46**, 10–18. 10.1111/j.1365-2664.2008.01600.x (2009).

[3] Lockwood, J. L., Hoopes, M. F. & Marchetti, M. P. *Invasion Ecology* 2nd edn. (Wiley-Blackwell, 2013).

[4] Bellard, C., Cassey, P. & Blackburn, T. M. Alien species as a driver of recent extinctions. *Biol. Lett.* **12**, 20150623. 10.1098/rsbl.2015.0623 (2016). 26888913 10.1098/rsbl.2015.0623 PMC4780541

[5] Dehnen-Schmutz, K., Touza, J., Perrings, C. & Williamson, M. A century of the ornamental plant trade and its impact on invasion success. *Divers. Distrib.* **13**, 527–534 (2007).

[6] van Kleunen, M. et al. Global exchange and accumulation of non-native plants. *Nature* **525**, 100–103 (2015). 26287466 10.1038/nature14910

[7] Richardson, D. M. *Ecology and Biogeography of Pinus* (Cambridge University Press, 1998).

[8] Lavi, A., Perevolotsky, A., Kigel, J. & Noy-Meir, I. Invasion of *Pinus halepensis* from plantations into adjacent natural habitats. *Appl. Veg. Sci.* **8**, 85–92 (2005).

[9] Bonari, G., Acosta, A. T. R. & Angiolini, C. Mediterranean coastal pine forest stands: Understorey distinctiveness or not?. *For. Ecol. Manage.* **391**, 19–28 (2017).

[10] Kong, W. S. Species composition and distribution of native Korean conifers. *J. Korean Geogr. Soc.* **39**, 528–543 (2004).

[11] Kong, W. S. Biogeography of native Korean Pinaceae. *J. Korean Geogr. Soc.* **41**, 73–93 (2006).

[12] Choi, W. I. et al. Changes in major insect pests of pine forests in Korea over the last 50 years. *Forests* **10**, 692. 10.3390/f10080692 (2019).

[13] Dixon, A. F. G. *Aphid Ecology* 2nd edn. (Chapman & Hall, 1998).

[14] Loxdale, H. D. Rapid genetic changes in natural insect populations. *Ecol. Entomol.* **35**, 155–164 (2009).

[15] Figueroa, C. C., Fuentes-Contreras, E., Molina-Montenegro, M. A. & Ramírez, C. C. Biological and genetic features of introduced aphid populations in agroecosystems. *Curr. Opin. Insect Sci.* **26**, 63–68 (2018). 29764662 10.1016/j.cois.2018.01.004

[16] Favret, C. (ed) & Aphid Taxon Community (eds). *Blackman & Eastop’s Aphids on the World’s Plants*, version 1.0. Available at: https://aphidsonworldsplants.info (Accessed 3 May 2025).

[17] Guo, Y. et al. Aphid viruses: a brief view of a long history. *Front. Insect Sci.* **2**, 846716. 10.3389/finsc.2022.846716 (2022). 38468755 10.3389/finsc.2022.846716 PMC10926426

[18] Ivanauskas, A. et al. New genetically distinct phytoplasmas and insect carriers associated with pine tree disease revealed by a survey in the Curonian Spit, Lithuania. *Can. J. For. Res.* **52**, 201–208 (2022).

[19] Kanturski, M. et al. Morphological and molecular insights into the invasive strawberry aphid *Chaetosiphon fragaefolii* – a critical pest and virus vector new to Poland. *J. Plant. Prot. Res.* **65**(4), 2 (2025).

[20] Normark, B. B. Molecular systematics and evolution of the aphid family Lachnidae. *Mol. Phylogenet. Evol.* **14**, 131–140 (2000). 10631047 10.1006/mpev.1999.0699

[21] Favret, C. & Voegtlin, D. J. A revision of the *Cinara* species (Hemiptera: Aphididae) of the United States pinyon pines. *Ann. Entomol. Soc. Am.* **97**, 1165–1197 (2004).

[22] Lee, M. et al. First record of *Cinara todocola* (Hemiptera: Aphididae) on endangered Christmas tree in South Korea: morphology, biology, and global invasion potential. *Sci. Rep.* **15**, 6691. 10.1038/s41598-025-91072-2 (2025). 40000720 10.1038/s41598-025-91072-2 PMC11861255

[23] Lee, M. et al. First record and integrative analysis of the invasive aphid *Cinara pilicornis* in South Korea. *Sci. Rep.* **15**, 29075. 10.1038/s41598-025-12656-6 (2025). 40781460 10.1038/s41598-025-12656-6 PMC12334602

[24] Chen, R. et al. An aphid lineage maintains a bark-feeding niche while switching to and diversifying on conifers. *Cladistics* **32**, 555–572 (2016). 34740301 10.1111/cla.12141

[25] Wieczorek, K. et al. Adapting to change: exploring the distribution dynamics of the alien and potentially invasive aphid species *Cinara curvipes* (Hemiptera: Aphididae) in the context of global warming. *Eur. Zool. J.* **92**, 258–279. 10.1080/24750263.2024.2449152 (2025).

[26] Ciesla, W. The cypress aphid, *Cinara cupressi* (Buckton) in Africa. In *Exotic Aphid Pests of Conifers: A Crisis in African Forestry* Vol. 160 (FAO, 1991).

[27] Murphy, S. T. et al. Status and impact of invasive conifer aphid pests in Africa. In: Nair KSS (eds) *Impact of Diseases and Insect Pests in Tropical Forests*, 289–297 (1996).

[28] Lowe, S., Browne, M., Boudjelas, S. & De Poorter, M. *100 of the World’s Worst Invasive Alien Species* (ISSG, IUCN, 2000).

[29] Montalva, C. et al. The cypress aphid in Chile: a review of the current situation and preliminary data of the biological control. *Bosque* **31**, 81–88. 10.4067/S0717-92002010000200002 (2010).

[30] Wieczorek, K., Świątek, P. & Durak, R. Influence of selected biogenic amines on development and demographic parameters of a temperate population of *Cinara* (*Cupressobium*) *cupressi* (Hemiptera, Aphididae). *Arthropod-Plant Interact.* **15**, 583–593 (2021).

[31] Seo, H. Y. A taxonomic study on the Korean Lachnidae (Homoptera, Aphidoidea). PhD thesis, Chonbuk National University, 202 pp. (1994).

[32] Hernández, R. & Rodríguez, M. Identification of aphids on *Pinus caribaea* Morelet and *Pinus cubensis* Grisebach, in Topes de Collantes. *Centro Agrícola* **12**, 11–14 (1985).

[33] Lazzari, S. M. N. & Zonta-de Carvalho, R. C. Aphids (Homoptera: Aphididae: Lachninae: Cirarini) on *Pinus* spp. and *Cupressus* sp. in Southern Brazil. In: *Proc. XXI Int. Congr. Entomol.*, 493 (2000).

[34] Delfino, M. A. & Binazzi, A. Further data on conifer aphids from Argentina (Aphididae Lachninae Eulachnini). *Redia* **88**, 3–7 (2005).

[35] Villalobos Muller, W., Pérez Hidalgo, N., Mier Durante, M. P. & Nieto Nafría, J. M. Aphididae (Hemiptera: Sternorrhyncha) from Costa Rica, with new records for Central America. *Boln. Asoc. Esp. Ent.* **34**, 145–182 (2010).

[36] Voegtlin, D., Villalobos, W., Sánchez, M. V., Saborío-R., G. & Rivera, C. A guide to the winged aphids (Homoptera) of Costa Rica.. *Rev. Biol. Trop.* **51**, 1–22 (2003). 15260169

[37] Tissot, A. N. Notes on the lachnini of Florida with descriptions of two new species (Homoptera: Aphididae). *Fla. Entomol.* **22**, 33–48 (1939).

[38] Fox, R. C. & Griffith, K. H. Pine seedling growth loss caused by cinaran aphids in South Carolina. *J. Ga. Entomol. Soc.* **12**, 29–34 (1977).

[39] Pepper, J. O. & Tissot, A. N. Pine-feeding species of *Cinara* in the eastern United States (Homoptera: Aphididae). *Fla. Agric. Exp. Stn. Monogr. Ser.* **3**, 160 (1973).

[40] Smith, C. F. & Parron, C. S. *An annotated list of Aphididae (Homoptera) of North America*. Tech. Bull. 255, North Carolina Agric. *Exp. Stn.*, 428 pp. (1978).

[41] Favret, C. et al. Actual and inferred checklist of the aphids (Hemiptera: Aphididae) of the Great Smoky Mountains National Park, with attendant ant and host plant associations. *Proc. Entomol. Soc. Wash.* **112**, 381–403. 10.4289/0013-8797.112.3.381 (2010).

[42] Jousselin, E. et al. Is ecological speciation a major trend in aphids? Insights from a molecular phylogeny of the conifer-feeding genus *Cinara*. *Front. Zool.* **10**, 56. 10.1186/1742-9994-10-56 (2013). 24044736 10.1186/1742-9994-10-56 PMC3848992

[43] del Rio Mora, A. A. & Voegtlin, D. J. Algunas observaciones sobre áfidos de importancia forestal en el campo experimental forestal “Barranca de Cupatitizio”, Uruapán, Michoacán. *Cienc For* **13**, 75–88 (1988).

[44] Zhang, G., Zhang, W. & Zhong, T. Studies on Chinese species of *Cinara* Curtis and descriptions of new species (Homoptera: Lachnidae). *Sinozoologia* **10**, 121–141 (1993).

[45] Zhang, G., Chen, X., Zhong, T. & Li, J. Lachnidae. In: *Fauna of Agricultural and Forestry Aphids of Northwest China*, 182–217 (1999).

[46] Durak, R. The overwintering strategy of the anholocyclic aphid *Cinara tujafilina*. *Physiol. Entomol.* **39**, 313–321 (2014).

[47] Patti, J. H. & Fox, R. C. Seasonal occurrence of *Cinara* spp. and *Essigella pini* Wilson on loblolly pine, *Pinus taeda* L. *J. Ga. Entomol. Soc.* **16**, 96–105 (1981).

[48] Fedde, G. F. The pine aphid complex of the genus *Cinara* in the Appalachian Highlands region of South Carolina. MSc thesis, Clemson University, 143 pp. (1965).

[49] Fedde, G. F. Comparative analyses of systematic procedures and biological characteristics relating to pine aphids of the genus *Cinara* of western South Carolina. PhD thesis, Clemson University, 152 pp. (1967).

[50] Messing, R. H. et al. Invasive aphids attack native Hawaiian plants. *Biol Invasions* **9**, 601–607 (2007).

[51] Cœur d’Acier, A., Pérez Hidalgo, N. & Petrović-Obradović, O. Aphids (Hemiptera, Aphididae). *Alien terrestrial arthropods of Europe. BioRisk* **4**, 435–474 (2010).

[52] Georgiev, G. et al. First record of *Cinara* (*Cinara*) *cedri cedri* Mimeur (Hemiptera Aphididae) in Bulgaria. *Redia* **104**, 161–165 (2021).

[53] Yu, G. Y. & Wang, H. First record of *Cinara cedri* Mimeur (Hemiptera, Aphididae, Lachninae) on *Cedrus deodara* in Beijing, China. *J. Environ. Entomol.* **36**, 260–263 (2014).

[54] Binazzi, F., Sabbatini Peverieri, G. & Roversi, P. F. First record in Cyprus of *Cinara* (*Cinara*) *cedri* Mimeur (Aphididae Lachninae) on *Cedrus brevifolia* (Hooker Fil.) Henry. *Redia* **98**, 151–154 (2015).

[55] Jurk, M., Poljaković-Pajnik, L. & Jurk, D. The first record of *Cinara curvipes* (Patch, 1912) (Homoptera, Aphididae) in Slovenia and its possible economic impact. *Zbornik Gozd Lesar*. **88**, 21–29 (2009).

[56] Nozaki, T., Kobayashi, Y. & Shigenobu, S. First record of the cedar bark aphid, *Cinara cedri cedri* Mimeur, 1936 (Hemiptera: Aphidoidea) in Japan, and identification of infecting Wolbachia strains. *BioInvasions Records* **11**, 900–911. 10.3391/bir.2022.11.4.09 (2022).

[57] Lee, J. et al. One new record of the genus *Cinara* Curtis, 1835 (Hemiptera: Aphididae: Lachninae) from Korea. *J. Asia-Pac Biodivers.* **13**, 465–469 (2020). 10.1016/j.japb.2020.04.004

[58] Hałaj, R. & Osiadacz, B. On foreign land: The conquest of Europe by *Cinara curvipes* (Patch, 1912). *Dtsch. Entomol. Z.* **62**, 261–265 (2015).

[59] Oğuzoğlu, Ş. & Avci, M. Distribution, biology, morphology and damage of *Cinara cedri* Mimeur, 1936 (Hemiptera: Aphididae) in the Isparta Regional Forest Directorate. *Forestist* **69**, 1–10 (2019).

[60] Oğuzoğlu, Ş. & Avci, M. Natural enemies of *Cinara cedri* Mimeur 1936 (Hemiptera: Aphididae) in Cedar Forests in Isparta Regional Forest Directorate. *Kastamonu Univ. J. Fac.* **19**, 173–185 (2019).

[61] Havelka, J. & Starý, P. *Cinara splendens* (Hemiptera: Aphididae: Lachninae) – First Record in Palaearctic Region. *Forests* **11**, 911. 10.3390/f11090911 (2020).

[62] Emonnot, P., Gayraud, Y., Leclant, F. & Remaudière, G. Sur la présence en France de *Cedrobium laportei* Remaudière puceron nuisible au cèdre. *C R Acad. Agric. Fr* **53**, 966–972 (1967).

[63] Fabre, J. P. Sur la présence en France de *Cinara cedri* (Mimeur) puceron nuisible au Cèdre. *C R Acad. Agric. Fr* **62**, 771–775 (1976).

[64] Martin, J. The identification of common aphid pests of tropical agriculture. *Trop. Pest Manag*. **29**, 212–220 (1983).

[65] Chakrabarti, S., Medda, P. K. & Kanturski, M. Conifer-feeding aphids (Insecta: Hemiptera: Aphididae) of India, Bhutan and Nepal with descriptions of three new species of the genus *Cinara*. *Eur. Zool. J.* **87**, 659–687 (2020).

[66] Foottit, R. G., Maw, H. V., von Dohlen, C. D. & Hebert, P. D. N. Species identification of aphids (Insecta: Hemiptera: Aphididae) through DNA barcodes. *Mol. Ecol. Resour* **8**, 1189–1201 (2008). 21586006 10.1111/j.1755-0998.2008.02297.x

[67] Kumar, S., Stecher, G. & Tamura, K. MEGA7: Molecular Evolutionary Genetics Analysis version 7.0. *Mol. Biol. Evol.* **33**, 1870–1874 (2016). 27004904 10.1093/molbev/msw054 PMC8210823

[68] Minh, B. Q. et al. IQ-TREE 2: New models and efficient methods for phylogenetic inference in the genomic era. *Mol. Biol. Evol.* **37**, 1530–1534 (2020). 32011700 10.1093/molbev/msaa015 PMC7182206

[69] Lanfear, R. et al. PartitionFinder 2: new methods for selecting partitioned models of evolution for molecular and morphological phylogenetic analyses. *Mol. Biol. Evol.* **34**, 772–773 (2016). 10.1093/molbev/msw260 28013191

[70] Rambaut, A. FigTree, a Graphical Viewer of Phylogenetic Trees. Institute of Evolutionary Biology, Univ Edinburgh. http://tree.bio.ed.ac.uk/software/figtree/ (accessed. May 2025)

[71] Wieczorek, K. & Sawka-Gądek, N. DNA barcoding and molecular phylogenetics revealed a new cryptic bamboo aphid species of the genus *Takecallis* (Hemiptera: Aphididae). *Appl. Sci.* **13**, 7798. 10.3390/app13137798 (2023).

[72] Puillandre, N., Lambert, A., Brouillet, S. & Achaz, G. ABGD, automatic barcode gap discovery for primary species delimitation. *Mol. Ecol.* **21**, 1864–1877 (2012). 21883587 10.1111/j.1365-294X.2011.05239.x

[73] Puillandre, N., Brouillet, S. & Achaz, G. ASAP: assemble species by automatic partitioning. *Mol. Ecol. Resour.* **21**, 609–620 (2021). 33058550 10.1111/1755-0998.13281

[74] Zhang, J., Kapli, P., Pavlidis, P. & Stamatakis, A. A general species delimitation method with applications to phylogenetic placements. *Bioinformatics* **29**, 2869–2876 (2013). 23990417 10.1093/bioinformatics/btt499 PMC3810850

[75] Rozas, J. et al. DnaSP 6: DNA sequence polymorphism analysis of large data sets. *Mol. Biol. Evol.* **34**, 3299–3302 (2017). 10.1093/molbev/msx248 29029172

[76] Clement, M., Posada, D. & Crandall, K. A. TCS: a computer program to estimate gene genealogies. *Mol. Ecol.* **9**, 1657–1660 (2000). 11050560 10.1046/j.1365-294x.2000.01020.x

[77] Leigh, J. W. & Bryant, D. POPART: full-feature software for haplotype network construction. *Methods Ecol. Evol.* **6**, 1110–1116 (2015).

