# Supplementary material for: Integrative study of two invasive North American pine aphids (Cinara atlantica and C. watsoni) in South Korea
Source: Sci Rep. 2026 May 17;16:22345. doi: 10.1038/s41598-026-46921-z (PMC13377120; doi:10.1038/s41598-026-46921-z)

**Integrative study of two invasive North American pine aphids (*Cinara atlantica* and *C. watsoni*) in South Korea**

Minho Lee^1, 2^, Mariusz Kanturski^3^, Colin Favret^4^, and Seunghwan Lee^1, 2*^

^1^Insect Biosystematics Laboratory, Department of Agricultural Biotechnology, Seoul National University, Seoul 08826, Korea.

^2^Research Institute of Agricultural and Life Sciences, Seoul National University, Seoul 08826, Korea.

^3^Institute of Biology, Biotechnology and Environmental Protection, Faculty of Natural Sciences, University of Silesia in Katowice, Bankowa 9, 40–007 Katowice, Poland.

^4^Department of Biological Sciences, Université de Montréal, 4101 East Sherbrooke Street, Montréal, Quebec, H1X 2B2, Canada

**Supplementary Table 1.** Summary of information on *Cinara* species and one outgroup used in the present analyses.

| No. | Species | Acc. No. | Country | Locality | Host plant | Haplotype |
| --- | --- | --- | --- | --- | --- | --- |
| 01 | *Cinara watsoni* | EU701624 | USA | Hawaii | - | Hap_3 |
| 02 | *Cinara watsoni* | EU701625 | USA | Hawaii | - | Hap_3 |
| 03 | *Cinara watsoni* | KF649477 | USA | California | *Pinus radiata* | Hap_3 |
| 04 | *Cinara watsoni* | KF649479 | USA | California | *Pinus radiata* | Hap_3 |
| 05 | *Cinara watsoni* | KP869179 | USA | Georgia | *Pinus taeda* | Hap_3 |
| 06 | *Cinara watsoni* | KP869183 | USA | South Carolina | *Pinus taeda* | Hap_3 |
| 07 | *Cinara watsoni* | PX317258 | USA | Florida | *Pinus rigida* | Hap_3 |
| 08 | *Cinara watsoni* | PX317259 | USA | Florida | *Pinus rigida* | Hap_3 |
| 09 | *Cinara watsoni* | PX317260 | USA | North Carolina | *Pinus rigida* | Hap_3 |
| 10 | *Cinara watsoni* | PX317261 | Mexico | Puebla | *Pinus* sp. | Hap_3 |
| 11 | *Cinara watsoni* | PX317262 | Mexico | Puebla | *Pinus* sp. | Hap_3 |
| 12 | *Cinara watsoni* | PX317263 | South Korea | Icheon | *Pinus rigida* | Hap_3 |
| 13 | *Cinara watsoni* | PX317264 | South Korea | Icheon | *Pinus rigida* | Hap_3 |
| 14 | *Cinara watsoni* | PX317265 | South Korea | Icheon | *Pinus rigida* | Hap_3 |
| 15 | *Cinara watsoni* | PX317266 | South Korea | Icheon | *Pinus rigida* | Hap_3 |
| 16 | *Cinara watsoni* | PX317267 | South Korea | Icheon | *Pinus rigida* | Hap_3 |
| 17 | *Cinara watsoni* | PX317268 | South Korea | Icheon | *Pinus rigida* | Hap_3 |
| 18 | *Cinara watsoni* | PX317269 | South Korea | Icheon | *Pinus rigida* | Hap_3 |
| 19 | *Cinara watsoni* | PX317270 | South Korea | Icheon | *Pinus rigida* | Hap_3 |
| 20 | *Cinara watsoni* | PX317271 | South Korea | Seoul | *Pinus rigida* | Hap_3 |
| 21 | *Cinara watsoni* | PX317272 | South Korea | Gwangju | *Pinus taeda* | Hap_3 |
| 22 | *Cinara watsoni* | PX317273 | South Korea | Gwangju | *Pinus taeda* | Hap_3 |
| 23 | *Cinara watsoni* | PX317274 | South Korea | Gwangju | *Pinus taeda* | Hap_3 |
| 24 | *Cinara watsoni* | PX317275 | South Korea | Gwangju | *Pinus taeda* | Hap_3 |
| 25 | *Cinara watsoni* | PX317276 | South Korea | Gwangju | *Pinus taeda* | Hap_3 |
| 26 | *Cinara watsoni* | PX317277 | South Korea | Gwangju | *Pinus taeda* | Hap_3 |
| 27 | *Cinara watsoni* | PX317278 | South Korea | Gwangju | *Pinus taeda* | Hap_3 |
| 28 | *Cinara watsoni* | PX317279 | South Korea | Gwangju | *Pinus taeda* | Hap_3 |
| 29 | *Cinara atlantica* | EU701608 | USA | Hawaii | - | Hap_2 |
| 30 | *Cinara atlantica* | EU701609 | USA | Hawaii | - | Hap_2 |
| 31 | *Cinara atlantica* | HQ578880 | USA | North Carolina | - | Hap_1 |
| 32 | *Cinara atlantica* | HQ578882 | USA | Georgia | - | Hap_1 |
| 33 | *Cinara atlantica* | HQ578915 | USA | Georgia | - | Hap_1 |
| 34 | *Cinara atlantica* | HQ578886 | USA | South Carolina | - | Hap_1 |
| 35 | *Cinara atlantica* | GU668598 | USA | Virginia | - | Hap_1 |
| 36 | *Cinara atlantica* | KR044891 | USA | South Carolina | - | Hap_1 |
| 37 | *Cinara atlantica* | KR040621 | USA | North Carolina | - | Hap_1 |
| 38 | *Cinara atlantica* | HQ578875 | USA | South Carolina | - | Hap_1 |
| 39 | *Cinara atlantica* | HQ578921 | USA | West Virginia | - | Hap_1 |
| 40 | *Cinara atlantica* | PX317280 | USA | Tennessee | *Pinus virginiana* | Hap_1 |
| 41 | *Cinara atlantica* | PX317281 | USA | Tennessee | *Pinus strobus* | Hap_1 |
| 42 | *Cinara atlantica* | PX317282 | USA | North Carolina | *Pinus rigida* | Hap_1 |
| 43 | *Cinara atlantica* | PX317283 | USA | Tennessee | *Pinus strobus* | Hap_1 |
| 44 | *Cinara atlantica* | PX317284 | USA | Tennessee | *Pinus strobus* | Hap_1 |
| 45 | *Cinara atlantica* | PX317285 | USA | North Carolina | *Pinus rigida* | Hap_1 |
| 46 | *Cinara atlantica* | PX317286 | USA | Tennessee | *Pinus strobus* | Hap_1 |
| 47 | *Cinara atlantica* | PX317287 | USA | Alabama | *Pinus taeda* | Hap_1 |
| 48 | *Cinara atlantica* | PX317288 | USA | Tennessee | *Pinus virginiana* | Hap_1 |
| 49 | *Cinara atlantica* | PX317289 | USA | Florida | *Pinus rigida* | Hap_1 |
| 50 | *Cinara atlantica* | PX317290 | USA | Florida | *Pinus rigida* | Hap_1 |
| 51 | *Cinara atlantica* | PX317291 | South Korea | Icheon | *Pinus rigida* | Hap_1 |
| 52 | *Cinara atlantica* | PX317292 | South Korea | Icheon | *Pinus rigida* | Hap_1 |
| 53 | *Cinara atlantica* | PX317293 | South Korea | Icheon | *Pinus rigida* | Hap_1 |
| 54 | *Cinara atlantica* | PX317294 | South Korea | Icheon | *Pinus rigida* | Hap_1 |
| 55 | *Cinara atlantica* | PX317295 | South Korea | Icheon | *Pinus rigida* | Hap_1 |
| 56 | *Cinara atlantica* | PX317296 | South Korea | Icheon | *Pinus rigida* | Hap_1 |
| 57 | *Cinara atlantica* | PX317297 | South Korea | Seoul | *Pinus rigida* | Hap_1 |
| 58 | *Cinara atlantica* | PX317298 | South Korea | Icheon | *Pinus densiflora* | Hap_1 |
| 59 | *Cinara atlantica* | PX317299 | South Korea | Icheon | *Pinus densiflora* | Hap_1 |
| 60 | *Cinara atlantica* | PX317300 | South Korea | Seoul | *Pinus densiflora* | Hap_1 |
| 61 | *Cinara atlantica* | PX317301 | South Korea | Seoul | *Pinus densiflora* | Hap_1 |
| 62 | *Cinara pergandei* | EU701620 | USA | Idaho | - | Hap_4 |
| 63 | *Cinara pergandei* | EU701621 | Canada | British Columbia | - | Hap_5 |
| 64 | *Cinara pergandei* | EU701622 | Canada | Manitoba | - | Hap_6 |
| 65 | *Cinara pinea* | KF639329 | France | Languedoc-Roussillon | *Pinus* sp. | Hap_7 |
| 66 | *Cinara pinea* | KF639330 | France | Auvergne | *Pinus* sp. | Hap_8 |
| 67 | *Cinara pinea* | KF649342 | France | Auvergne | *Pinus* sp. | Hap_8 |
| 68 | *Cinara pinivora* | KR039219 | Canada | Alberta | - | Hap_10 |
| 69 | *Cinara pinivora* | KR039505 | Canada | Alberta | - | Hap_11 |
| 70 | *Cinara pinivora* | KF649501 | USA | Washington | *Pinus contorta* | Hap_9 |
| 71 | *Cinara taedae* | KP869182 | USA | - | *Pinus eliotti* | Hap_12 |
| 72 | *Lachnus tropicalis* | JQ916807 | outgroup | | | |

**Supplementary Figure 1.** Geographic distribution of populations, sample sizes, and COI haplotypes of *Cinara atlantica*. The basic map was obtained from a free map-providing site (https://d- maps. com)


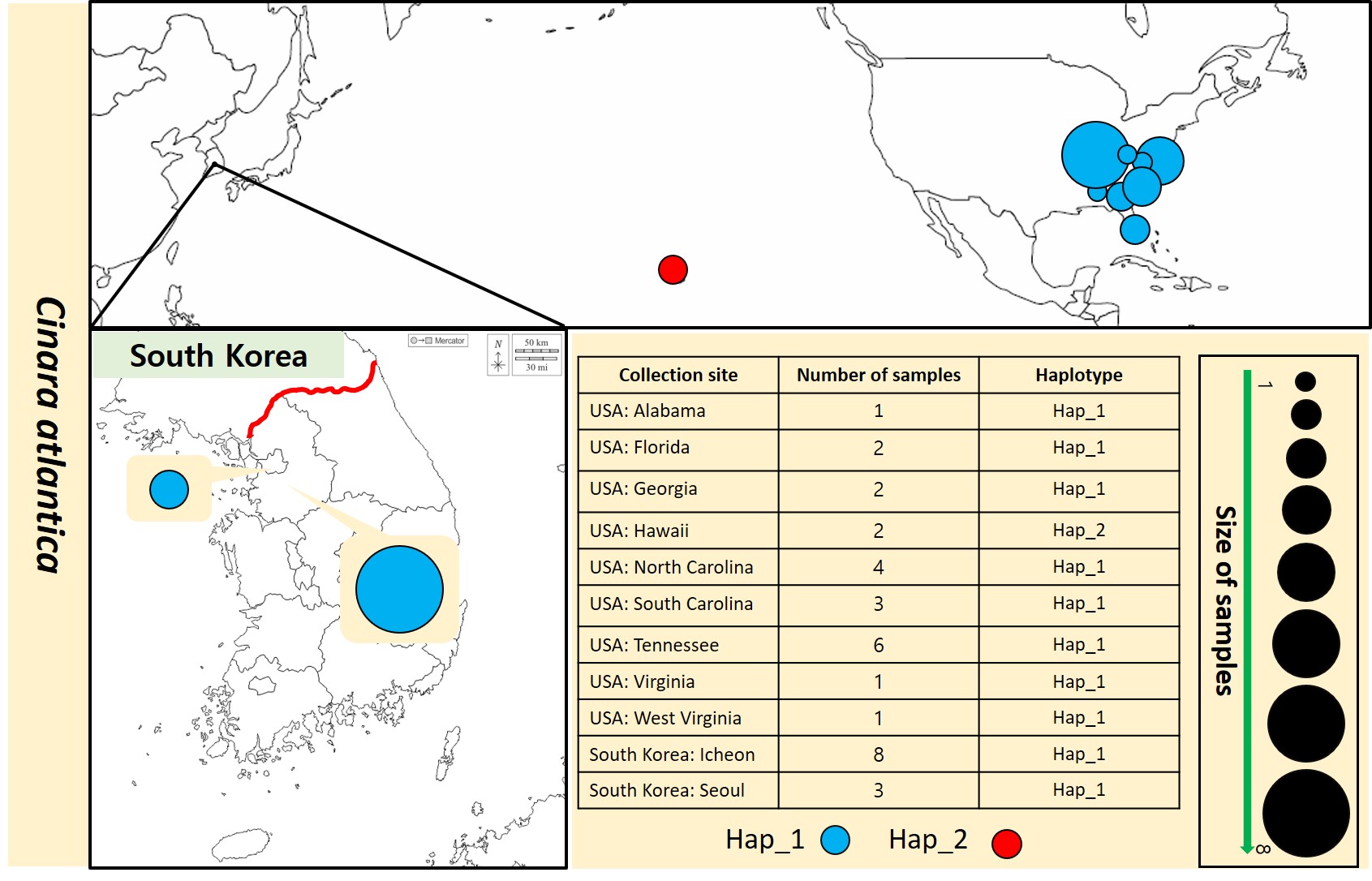


**Supplementary Figure 2.** Geographic distribution of populations, sample sizes, and COI haplotypes of *Cinara watsoni*. The basic map was obtained from a free map-providing site (https://d- maps. com)


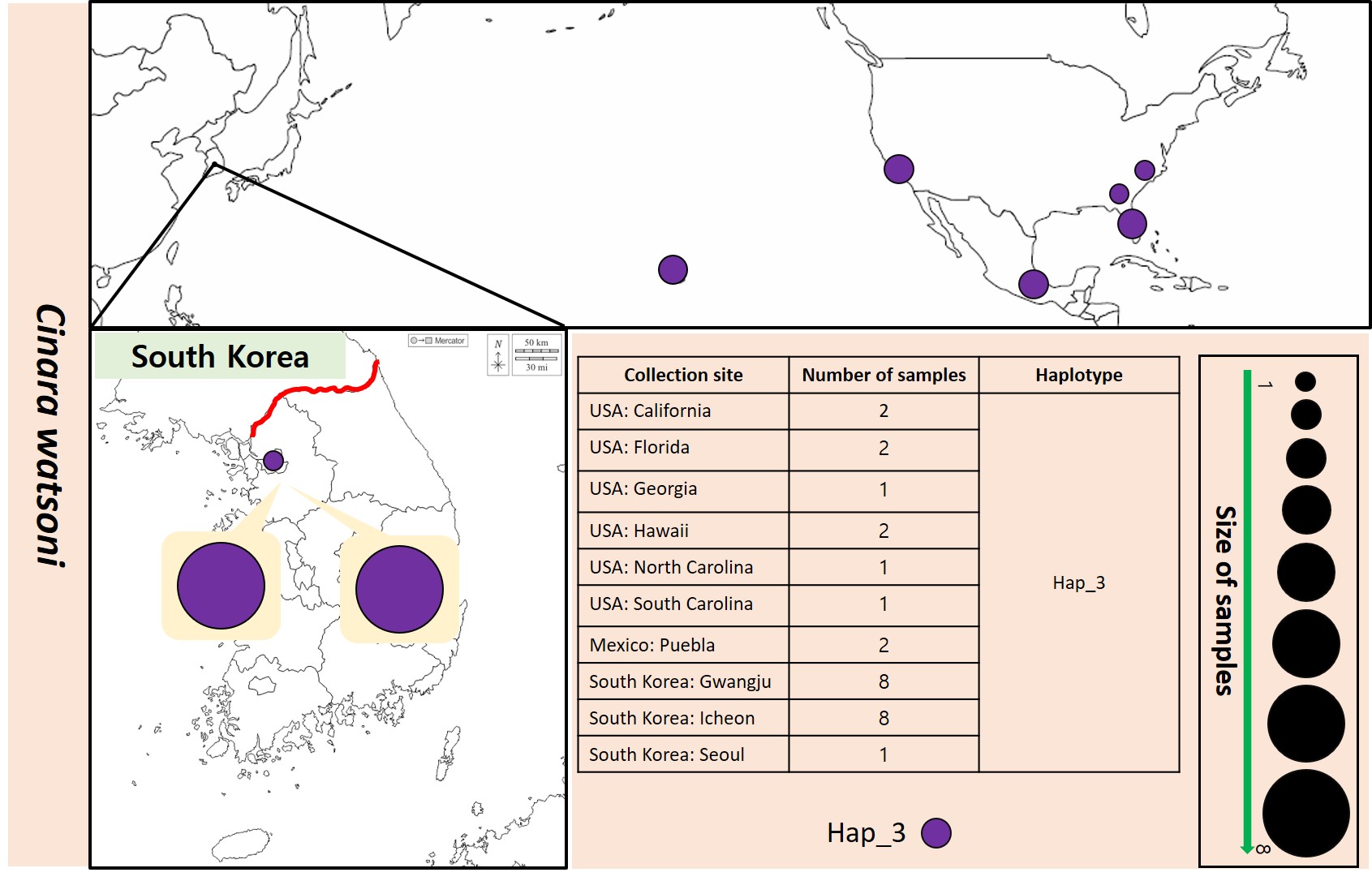

Supplement: Supplementary file 1 — Supplementary Material 1 [file 41598_2026_46921_MOESM1_ESM.docx]
